# *Eotaria citrica*, sp. nov., a new stem otariid from the “Topanga” formation of Southern California

**DOI:** 10.7717/peerj.3022

**Published:** 2017-02-23

**Authors:** Jorge Velez-Juarbe

**Affiliations:** 1 Department of Mammalogy, Natural History Museum of Los Angeles County, Los Angeles, CA, USA; 2 Department of Paleobiology, National Museum of Natural History, Washington, DC, USA

**Keywords:** Pinnipedia, Miocene, California, Otariidae, Taxonomy, Phylogenetic systematics

## Abstract

A new taxon of stem otariid, *Eotaria citrica* sp. nov., is described from the upper Burdigalian to lower Langhian “Topanga” formation of Orange County, California. The new species is described from mandibular and dental remains that show a unique combination of plesiomorphic and derived characters. Specifically, it is characterized by having trenchant and prominent paraconid cusps in p3–m1, lingual cingula of p2–4 with faint crenulations, premolars and molars with vestigial metaconid, bilobed root of m2 and a genial tuberosity located under p3. Furthermore, additional material of the contemporaneous *Eotaria crypta* is described, providing new information on the morphology of this taxon. Both species of *Eotaria* represent the earliest stem otariids, reinforcing the hypothesis that the group originated in the north Eastern Pacific Region. At present, the “Topanga” Fm. pinniped fauna includes *Eotaria citrica*, *Eotaria crypta*, the desmatophocid *Allodesmus* sp., the odobenids *Neotherium* sp., *Pelagiarctos* sp. and includes the oldest records of crown pinnipeds in California. Overall this pinniped fauna is similar to the nearly contemporaneous Sharktooth Hill bonebed. However, unambiguous records of *Eotaria* are still missing from Sharktooth Hill. This absence may be due to taphonomic or paleoenvironmental factors. The new “Topanga” record presented here was integrated into an overview of the late Oligocene through early Pleistocene pinniped faunas of Southern California. The results show an overall increase in body size over time until the Pleistocene. Furthermore, desmatophocids were the largest pinnipeds during the middle Miocene, but were extinct by the beginning of the late Miocene. Odobenids diversified and became the dominant pinnipeds in late Miocene through Pleistocene assemblages, usually approaching or exceeding 3 m in body length, while otariids remained as the smallest taxa. This pattern contrasts with modern assemblages, in which the phocid *Mirounga angustirostris* is the largest pinniped taxon in the region, odobenids are extinct and medium and small size ranges are occupied by otariids or other phocids.

## Introduction

The early to middle Miocene “Topanga” Formation of Orange County, California is known for its diverse marine tetrapod fauna (Domning, 1978; Raschke, 1984; Howard & Barnes, 1987; Aranda-Manteca, Domning & Barnes, 1994; see review by Boessenecker & Churchill (2013), regarding the use of “Topanga” vs Topanga for this unit). In addition to marine birds, sirenians and cetaceans, several pinnipeds have been recognized from this formation, including the stem otariid *Eotaria crypta* Boessenecker & Churchill, 2015, the odobenids *Pelagiarctos* Barnes, 1988, and *Neotherium* Kellogg, 1931, as well as remains of the desmatophocid *Allodesmus* Kellogg, 1922 (Raschke, 1984; Howard & Barnes, 1987; Boessenecker & Churchill, 2013; Garibay, Velez-Juarbe & Parham, 2016). The presence of *Pelagiarctos*, *Neotherium* and *Allodesmus*, as well as some of the marine birds and cetaceans, hints at taxonomic similarities between the “Topanga” Fm. and the nearly coeval Sharktooth Hill bonebed of the Round Mountain Silt (Howard & Barnes, 1987). However, at least some of the “Topanga” pinnipeds may represent different species than the ones found at Sharktooth Hill (Boessenecker & Churchill, 2013; Garibay, Velez-Juarbe & Parham, 2016), while other “Topanga” pinnipeds, such as *Eotaria crypta*, seem to be absent from Sharktooth Hill.

Here, I describe a second species of the stem otariid *Eotaria*, based on mandibular and dental remains from the upper part of the “Topanga” Formation at Oso Dam in Orange County, California. Both species of *Eotaria* share a combination of derived and plesiomorphic characteristics that set them apart from all other known otariids and both seem to be absent from the Sharktooth Hill bonebed. These differences in the pinniped fauna between these two nearly coeval formations may be the result of temporal, environmental or taphonomic differences between these two units.

Furthermore, the relatively good record of fossil marine mammals in Southern California (Barnes, 1976) allows for a comparison of the “Topanga” pinnipeds with faunas from older and younger deposits. The results of this comparison show some interesting changes in the taxonomic composition as well as patterns of body size changes in the pinniped faunas of the region across the last 25 million years.

## Materials and Methods

### Phylogenetic analysis

A phylogenetic analysis was performed using the character matrix from Boessenecker & Churchill (2015), adding *Eotaria citrica*, new character scoring for *Eotaria crypta* based on LACM 159981 and *Pithanotaria starri* Kellogg, 1925, based on LACM 22449, 52773, 115153 and 115677. Additionally, characters 16 and 61 were rescored for *Thalassoleon mexicanus* Repenning & Tedford, 1977, based on the holotype and referred specimens (Deméré & Berta, 2005) (Data S1); most characters were treated as unordered with the exception of those that had a polymorphic state (following Boessenecker & Churchill, 2015). In addition a backbone constraint tree of crown Otariidae was used, as their relationships are fairly stable based on molecular, morphological and combined analyses (Yonezawa, Kohno & Hasegawa, 2009; Churchill, Boessenecker & Clementz, 2014). The matrix was analyzed using PAUP* (Swofford, 2002), by doing a heuristic search using the tree bisection–reconnection (TBR) algorithm. Statistical support was done by doing 1,000 bootstrap replicas and searching for successive longer trees to calculate decay indices. The final matrix is available in .nex format in Supplemental Information (Data S1).

### Principal component analysis

A principal component analysis (PCA) was performed to quantitatively assess whether the differences in overall size between species of *Eotaria* were the result of sexual dimorphism. For the analysis, ramus width and height, total length of the mandible as well as the diameter of the canine of *Eotaria citrica*, *Eotaria crypta* and a subset of *Zalophus californianus* (Lesson, 1828) (22 females, 32 males), *Callorhinus ursinus* (Linnaeus, 1758) (five females, one male) and *Eumetopias jubatus* (von Schreber, 1776) (five females, two males) were compared (Table S1). Analyses were performed in R (R Development Core Team, 2012) using the function “princomp.” Additionally, the medoids and distance between medoids of the female and male groups were calculated using the functions “pam” and “dist,” respectively, for comparison with the distance between both species of *Eotaria* (Table S2).

### Nomenclature acts

The electronic version of this article in portable document format will represent a published work according to the International Commission on Zoological Nomenclature (ICZN), and hence the new names contained in the electronic version are effectively published under that code from the electronic edition alone. This published work and the nomenclatural acts it contains have been registered in ZooBank, the online registration system for the ICZN. The ZooBank life science identifiers (LSIDs) can be resolved and the associated information viewed through any standard web browser by appending the LSID to the prefix “http://zoobank.org/”. The LSID for this publication is: urn:lsid:zoobank.org:pub:AA9D04DC-A79E-49AC-85AE-744D7BA39D6D. The online version of this work is archived and available from the following digital repositories: PeerJ, PubMed Central and CLOCKSS.

### Specimens observed

*Allodesmus* sp. (LACM 125969, 126199, 160016; OCPC 5670); *Allodesmus kelloggi* Mitchell, 1966 (LACM 4320); *Callorhinus gilmorei* Berta & Deméré, 1986 (LACM 115253, 4323; SDSNH 25176, 25554); *Callorhinus ursinus* (LACM 51351, 51353, 51354, 51356, 51357, 51545, 52331, 52341, 523412, 86090); *E. jubatus* (LACM 616, 620, 21443, 39651, 51173, 52311–52313, 52315, 97334); *Gomphotaria pugnax* Barnes & Raschke, 1991 (LACM 105151, 121508; LC 7750); *Neotherium mirum* Kellogg, 1931 (LACM 81665, 123000, 123002, 131950, 134393); Odobenidae sp. 1 (LACM 123282); Odobenidae sp. 2 (LACM 122444); Odobenidae sp. 3 (LACM 4324, 17588); Odobenidae sp. 4 (LACM 118967); Odobenidae sp. 5 (LACM 150922); Otariidae (OCPC 1893, 1894); *Pelagiarctos* sp. (LACM 118601); *Pelagiarctos thomasi* Barnes, 1988 (LACM 121501); Pinnipedia indet. (LACM 127710); *P. starri* (LACM 22445, 22449, 31202, 37582, 122620, 122621, 52773, 115153, 115677, 117687); cf. *Proneotherium* (LACM 128412); *Thalassoleon* sp. (LACM 128005, 150914); *Thalassoleon inouei* Kohno, 1992 (LACM 131942 [cast of CBMPV 087]); *Thalassoleon mexicanus* (LACM 149498 [cast of IGCU 902]; SDSNH 65163, 65172); *Z. californianus* (LACM 343, 8584, 8585, 9337, 22557, 22999, 23000, 31275, 31360, 39652–39655, 39661–39666, 43482, 51164, 51170, 51171, 51175, 51182, 51191, 51192, 51197, 51199, 51204, 51218, 51220, 51221, 51223, 51228, 51229, 51237, 52321, 51406, 52411, 52412, 52418, 54104, 54421, 54462, 54578, 54590, 54624, 84098, 86060, 91326–91329, 91332, 91334, 91761, 91857, 91889, 97236, 97240, 97517, 97520, 97569, 97576, 97578, 97581, 97586, 97588, 97597).

## Systematic Paleontology

CARNIVORA Bowdich, 1821
PINNIPEDIA Illiger, 1811
PAN-OTARIIDAE new clade name (pan-stem based version of Otariidae Gill, 1866)

**Definition:** “Pan-Otariidae” refers to the pans-tem that includes crown Otariidae, and all other taxa closer to it, than to *Phoca vitulina* Linnaeus, 1758, or *Odobenus rosmarus* Linnaeus, 1758.

**Subjective synonyms:** Otariidae Gill, 1866, *sensu* Berta, Sumich & Kovacs (2015).

*EOTARIA* Boessenecker & Churchill, 2015

**Type:** *Eotaria crypta* Boessenecker & Churchill, 2015.

**Included species:** *Eotaria crypta* Boessenecker & Churchill, 2015; *Eotaria citrica* sp. nov.

**Range:** Early to middle Miocene (upper Burdigalian to lower Langhian) of CA, USA.

**Emended diagnosis:** Small pinnipeds, with an estimated body length less than 1.5 m. Shares with all other known pan-otariids, reduction (or absence) of metaconid cusp and protoconid with concave posterior margin (c. 94[3]). Characterized by having molars and premolars that are longer than high and transversely narrow (c. 74[0]), well-developed, conical paraconid and hypoconid (c. 93[1]) and presence of m2 (c. 98[0]; also present in some *P. starri*; Fig. 6D).

**Figure 1:**
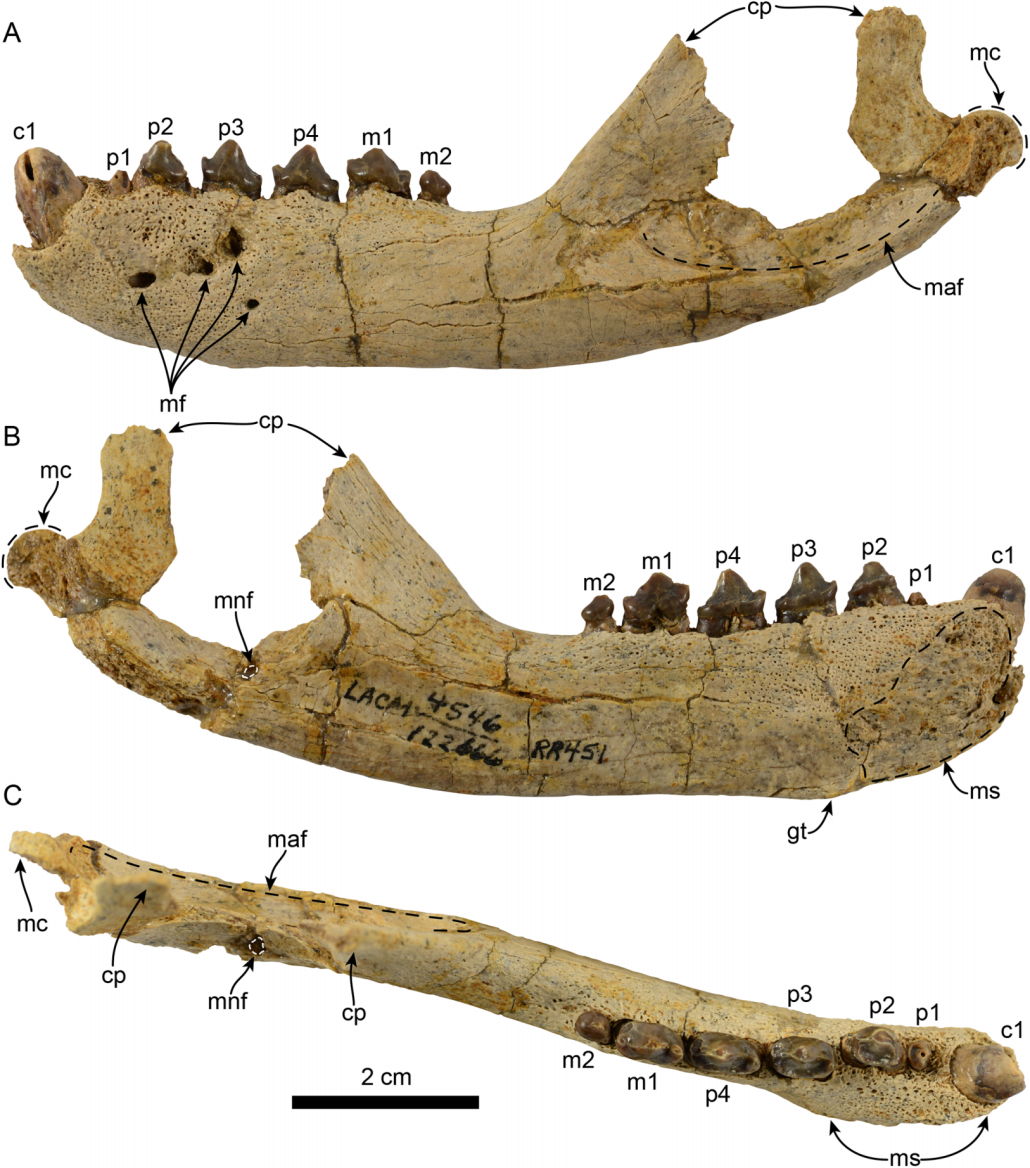
Mandible of *Eotaria citrica* sp. nov. (LACM 122666). Mandible in lateral (A), medial (B) and occlusal (C), views. Abbreviations: c1, first lower canine; cp, coronoid process; gt, genial tuberosity; m1–2, lower molars 1–2; maf, masseteric fossa; mc, mandibular condyle; mf, mental foramina; mnf, mandibular foramen; ms, mandibular symphysis; p1–4, lower premolars 1–4.

**Figure 2:**
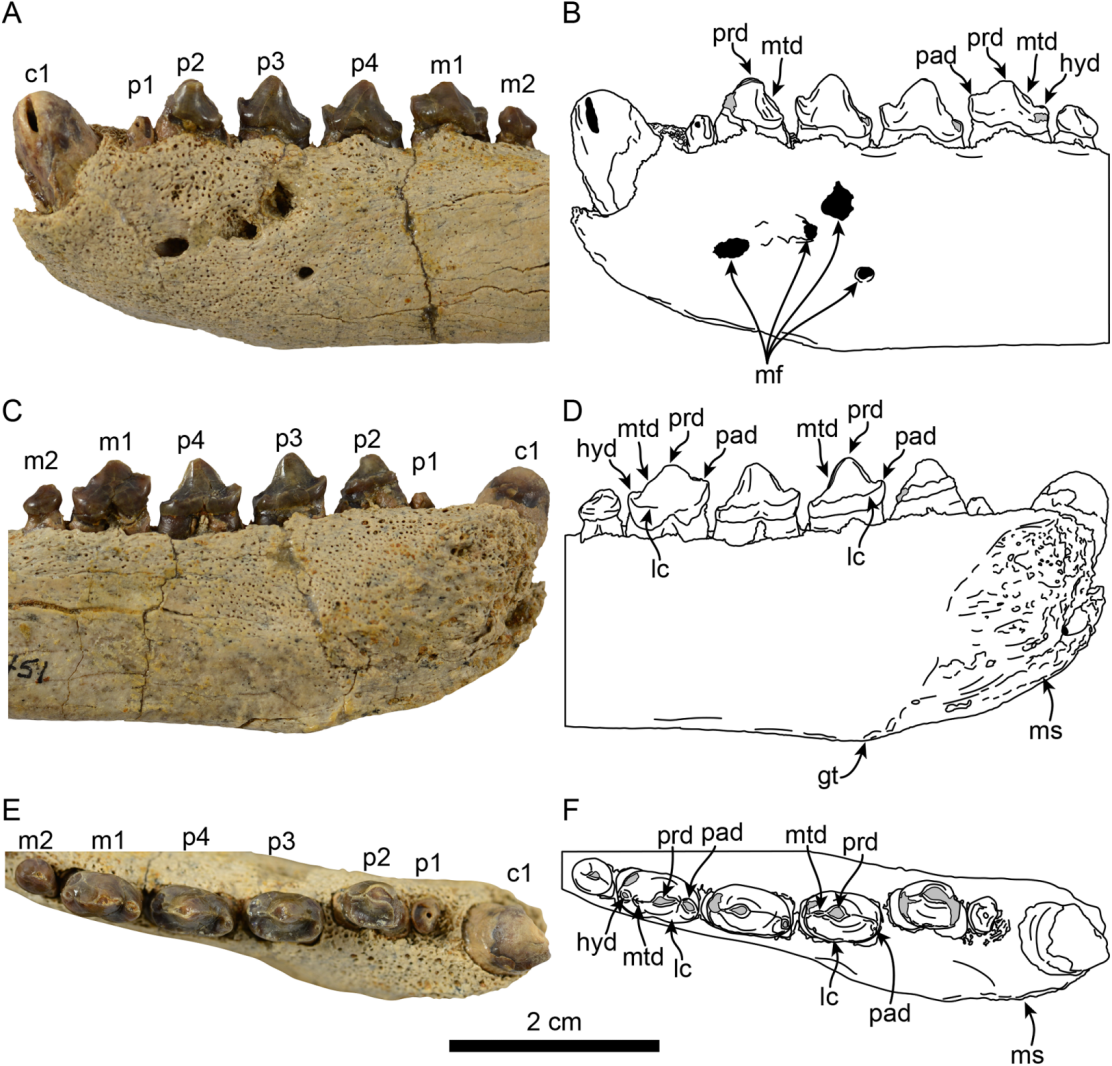
Dentition of *Eotaria citrica* sp. nov. (LACM 122666). Detail of teeth in lateral (A, B), medial (C, D) and occlusal (E, F), views. Abbreviations: c1, first lower canine; gt, genial tuberosity; hyd, hypoconid; lc, lingual cingulum; m1–2, lower molars 1–2; mf, mental foramina; ms, mandibular symphysis; mtd, metaconid; p1–4, lower premolars 1–4; pad, paraconid; prd, protoconid.

*EOTARIA CITRICA*, sp. nov.
(Figs. 1 and 2; Tables 1 and 2)

**Table 1:** Measurements (in mm) of mandibles of *Eotaria* spp. Measurements (in mm) of mandible of *Eotaria citrica* sp. nov. (LACM 122666) and *Eotaria crypta* (LACM 159981 and OCPC 5710) (following Boessenecker & Churchill, 2015).

	LACM 122666	LACM 159981	OCPC 5710^1^
Total length of mandible	115.10	95.76	–
Dorsoventral depth of mandible at p2	19.98	15.10	14.50
Dorsoventral depth of mandible at p3	20.70	14.08	14.33
Dorsoventral depth of mandible at p4	19.68	13.72	13.67
Dorsoventral depth of mandible at m1	19.76	13.10	13.63
Dorsoventral depth of mandible at m2	18.88	13.14	13.42
Distance between p2–3 crown apices	8.22	7.36	6.92
Distance between p3–4 crown apices	9.10	7.36	7.48
Distance between p4–m1 crown apices	8.02	6.68	6.42
Diastema between p2–3	1.32	1.14	0.90
Diastema between p3–4	1.52	1.14	0.96
Diastema between p4–m1	1.18	1.00	1.26
Length of coronoid process at base	44.74	33.72	–
Length of toothrow	49.96	41.80	–
Length of masseteric fossa	39.30	36.14	–

**Note:**

1 Measurements from Boessenecker & Churchill (2015).

**Table 2:** Measurements (in mm) of dentition of *Eotaria* spp. Measurements (in mm) of mandibular, canine and postcanine dentition of *Eotaria citrica* sp. nov. (LACM 122666) and *Eotaria crypta* (LACM 159981 and OCPC 5710) (following Boessenecker & Churchill, 2015).

	LACM 122666	LACM 159981	OCPC 5710^1^
c1: Anteroposterior length		4.74	–
c1: Height of crown		10.10	–
c1: Transverse width		4.84	–
p1: Anteroposterior length	2.54^2^	2.52^2^	–
p1: Height of crown	–	–	–
p1: Transverse width	3.42^2^	2.52^2^	–
p2: Anteroposterior length	5.80	5.68	5.59
p2: Height of crown	4.68	5.22	5.63
p2: Transverse width	4.38	3.40	4.33
p3: Anteroposterior length	7.56	6.66	6.65
p3: Height of crown	5.74	5.94	5.97
p3: Transverse width	4.68	3.93	3.82
p4: Anteroposterior length	8.12	6.64	6.73
p4: Height of crown	6.02	5.72	5.72
p4: Transverse width	4.58	3.58	3.70
m1: Anteroposterior length	7.72	6.76	6.83
m1: Height of crown	5.66	5.26	5.81
m1: Transverse width	4.42	3.70	3.67
m2: Anteroposterior length	3.98	2.10^2^	
m2: Height of crown	3.08	–	–
m2: Transverse width	3.26	2.10^2^	

**Notes:**

1 Measurement from Boessenecker & Churchill (2015).

2 Measurement of alveolus or root.

**Holotype:** LACM 122666, nearly complete left mandible, including c1, p2–4, m1–2. Collected by R. Raschke, December 28, 1978.

**Type locality and horizon:** LACM loc. 4546 (Upper Oso Dam), cobble horizon near top of “Topanga” Fm. exposed on east side of Oso Dam, Mission Viejo, Orange County, CA, USA; 33°39′30″N, 117°37′35″W (Howard & Barnes, 1987: Fig. 1; Whistler & Lander, 2003: Fig. 11.2). Other vertebrates from this horizon include: *Isurus hastalis*, dermochelyid and cheloniid turtles, *Puffinus* sp., *Puffinus priscus*, Sulidae indet., Anatinae indet., *Alcodes* aff. *A. ulnulus*, desmostylians, aff. *Neotherium* sp., *Allodesmus* sp., *Kentriodon* aff. *K. obscurus* and cetotheriid mysticetes (Raschke, 1984; Howard & Barnes, 1987).

**Formation:** “Topanga” formation (see review by Boessenecker & Churchill (2013), regarding the use of “Topanga” vs Topanga for this unit).

**Age:** Upper Burdigalian to lower Langhian (16.5–14.5 Ma). The age of the “Topanga” Fm. in Orange County, CA, USA was thoroughly discussed by Boessenecker & Churchill (2013, 2015), who concluded that the best reference for the overall age of this formation is the presence of Relizian to early Luisian foraminifera. However, the range used for these foraminiferal zones is herein updated based on Ogg, Ogg & Gradstein (2008).

**Range:** Early to middle Miocene (upper Burdigalian to lower Langhian) of CA, USA.

**Differential diagnosis:** Small sized pinniped, with an estimated body length of 1.40 m (based on mandible length and toothrow length formulas of Churchill, Clementz & Kohno, 2014). Differs from *Eotaria crypta* by having a more trenchant and prominent paraconid cusp in p3–m1, p2–4 with faintly crenulated distolingual edge of lingual cingula, vestigial metaconid present on the premolars, m2 with a bilobed root, a larger and more robust horizontal ramus (ratio of width/height of ramus at p4 = 0.45 in *Eotaria citrica*, 0.35 in *Eotaria crypta*), genial tuberosity located posterior to p2 (c. 72[2]; shared with the *Pithanotaria* + *Callorhinus* clade) and by having a p1 root with diameter smaller than the roots of the other premolars (c. 96[1]; shared with *Proterozetes ulysses* Barnes, Ray & Koretsky, 2006, and *E. jubatus*).

**Etymology:** The specific epithet derives from Latin *Citrus*, the genus of the orange fruit and namesake of Orange County, CA, USA.

**Description:** The description of the mandible is based solely on the holotype and only known specimen.

**Mandible:** The horizontal ramus is dorsoventrally slender, with dorsal and ventral edges straight (c. 65[0]) and nearly parallel. The mandibular symphysis is oval in outline, with its long axis oriented anterodorsal to posteroventral (Figs. 1 and 2). On the lateral surface there are four mental foramina at the level between p1 and p3; these are relatively small, ranging in size from 1 to 2 mm in diameter. The genial tuberosity is relatively inconspicuous and located in line with the anterior root of p3 (c. 66[0], 72[2]); it is at this level that the horizontal ramus reaches its greater dorsoventral depth (c. 71[1]) (Table 1). Posterior to the genial tuberosity, the ventral border of the horizontal ramus is nearly straight, curving posterodorsally at the level of the nearly inconspicuous insertion site for musculus digastricus (c. 67[0]). The base of the coronoid process is anteroposteriorly short, measuring less than half of the total length of the mandible (c. 70[0]). The anterior edge of the coronoid process slopes posterodorsally at about 120° from the horizontal ramus, while its posterior edge, although incompletely preserved, seems to have been vertical. The masseteric fossa is long (c. 73[0]) and relatively deep; its ventral edge is delineated by a ridge that begins at a level just posterior to the anterior edge of the coronoid and extends posteriorly to the base of the mandibular condyle. The mandibular condyle is incompletely preserved medially and laterally, the articular surface is oriented posterodorsally, located at a level above the toothrow and relatively close to the posterior edge of the coronoid process. The mandibular foramen opens posterodorsally and is relatively small (∼2 mm in diameter); it is located about 6 mm from the ventral border of the mandible. The alveolar row is incompletely preserved anteriorly, as there are no incisor alveoli preserved and only a small (∼1 mm in diameter) foramen located ventromedial to the canine which may have been part of the lateral-most incisor alveoli. In dorsal view the postcanine dentition are arranged in a medially convex arch.

**Dentition:** The preserved dentition includes complete or partially complete crowns of c1, p1–4, m1–2; the lower toothrow is long relative to the total length of the mandible (c. 90[0]). The canine is incompletely preserved, but it is relatively large, conical and oriented anterodorsally (Figs. 1 and 2). Overall the postcanine teeth have crowns that are transversely narrow and longer than high (c. 74[0]) and none of them have buccal cingula (c. 82[0]). The postcanines are nearly all double-rooted (c. 92[0]), with the exception of p1 and m2 which has a bilobed root.

The first premolar is single-rooted; its crown is broken near the base, providing no information about its morphology, except that is was likely the smallest of the postcanine teeth (c. 95[0], 96[1]). Premolars 2–4 are longer than high with nearly straight lingual cingula (c. 99[2]) and no buccal cingula. The lingual cingula arise from the mesiolingually located paraconid, bordering the lingual edge of the teeth and distally reaching the low, nearly inconspicuous hypoconid; p2–4 show faint crenulations along the distolingual edge of the cingulum. The paraconid is connected to the main cusp, formed by the protoconid, via a ridge that descends mesially from the apex of the latter. This ridge gives the mesial edge of the protoconid a convex outline, while its distal edge is shallowly concave with a vestigial metaconid (c. 94[3]). The first molar is characterized by being longer than high, with a protoconid noticeably lower than those of premolars 2–4. In m1 the paraconid and hypoconid occupy a similar position as in premolars 3–4, however they are slightly larger and more conical (c. 93[1]), while the lingual cingulum is less conspicuous. On the distal edge between the apex of the protoconid and the hypoconid is a low, nearly indistinct metaconid (c. 94[3]) (Fig. 2), reminiscent of the condition of *Eotaria crypta* (Boessenecker & Churchill, 2015). The m2 is present (c. 98[0]) and noticeably smaller than m1 and p2–4, its crown is nearly conical, consisting solely of the protoconid and no cingula, similar to m2 of *N. mirum*.

**Figure 3:**
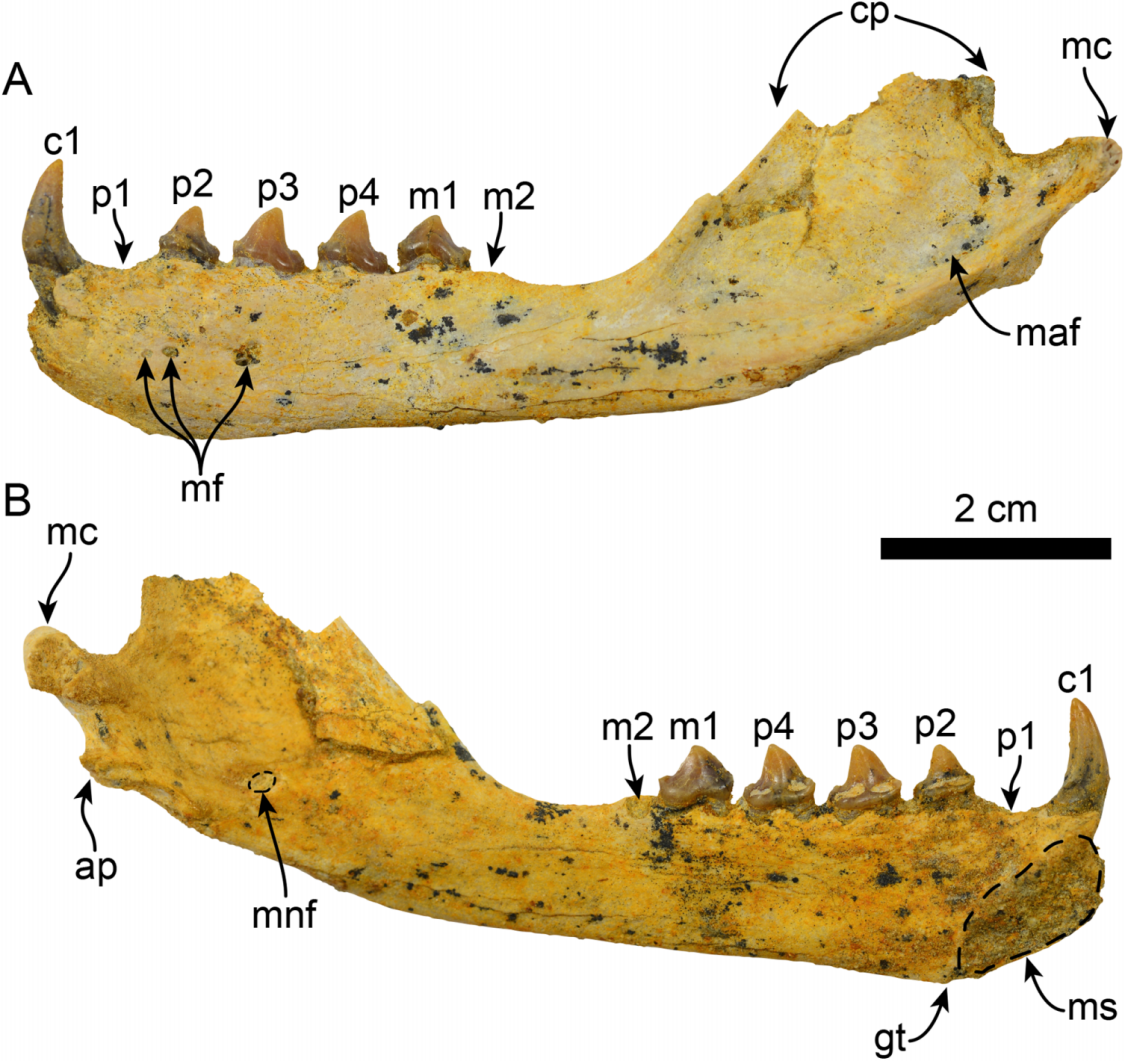
Mandible of *Eotaria crypta* (LACM 159981). Mandible in lateral (A) and medial (B), views. Abbreviations: ap, angular process; c1, first lower canine; cp, coronoid process; gt, genial tuberosity; m1–2, lower molars 1–2; maf, masseteric fossa; mc, mandibular condyle; mf, mental foramina; mnf, mandibular foramen; ms, mandibular symphysis; p1–4, lower premolars 1–4.

*EOTARIA CRYPTA* Boessenecker & Churchill, 2015
(Fig. 3; Tables 1 and 2)

**Holotype:** OCPC 5710, partial right mandible with p2–4 and m2 alveolus (Boessenecker & Churchill, 2015).

**Type locality and horizon:** Mission Viejo, Orange County, CA, USA.

**Age:** Upper Burdigalian to lower Langhian (16.5–14.5 Ma) (Boessenecker & Churchill, 2015; age of foraminiferal zones corrected following Ogg, Ogg & Gradstein, 2008).

**Newly referred material:** LACM 159981, nearly complete left mandible, including c1, p2–4 and m1; missing the apex of the coronoid process and the medial half of the mandibular condyle, p1 and m2.

**Locality horizon and age:** LACM loc. 6064, Mission Viejo, Orange County, CA, USA. The specimen comes from a fine to medium sandstone unit of the “Topanga” formation, upper Burdigalian to lower Langhian (16.5–14.5 Ma) in age.

**Differential diagnosis:** Small sized pinniped, with an estimated body length of 1.25 m (based on mandible length and toothrow length formulas of Churchill, Clementz & Kohno, 2014). Differing from *Eotaria citrica* by the following combination of characters: p3–4 and m1 with less trenchant paraconid, p2–4 with smooth lingual cingula, absence of metaconid cusp in the premolars, m2 root rounded, genial tuberosity at the level of p2 (c. 72[0]; shared with the *Thalassoleon* + *Eumetopias* clade) and p1 root larger (c. 96[0]; shared with most other pan-otariids).

**Description:** The description is based on the more complete newly referred specimen; for a thorough description of the holotype, see Boessenecker & Churchill (2015).

The horizontal ramus is relatively slender and mediolaterally thin. The dorsoventrally deepest portion is at the level of the genial tuberosity (c. 71[1]), which as in OCPC 5710 lies at the level below p2, while it is thinner between the m2 alveolus and the base of the coronoid process. Medially, the mandibular symphysis is more rectangular, in contrast to the more ovoid symphysis in *Eotaria citrica*, its long axis oriented anterodorsally–posteroventrally. On its lateral surface there are three mental foramina ranging in size from 0.6 to 0.9 mm in diameter, with the posteriormost being the largest and located at the level of p3. The ventral edge of the horizontal ramus is relatively straight (c. 65[0]), diverging posterodorsally at the level of the base of the coronoid process; the digastric insertion is nearly indistinct, marked only by a small rugose area (c. 67[0]). As preserved, the coronoid process seems to have been relatively low and anteroposteriorly narrow (c. 70[0]), with its anterior edge sloping posterodorsally at about 130° from the horizontal. On the lateral surface, the masseteric fossa is long (c. 73[0]), relatively shallow anteriorly and becoming deeper posteriorly, with its posteroventral edge forming a shelf that continues posteriorly towards the base of the mandibular condyle. The mandibular condyle is incompletely preserved but seems to have been oriented posterodorsally and is located at a level above the toothrow. On the medial side of the posterior half of the mandible, the mandibular canal opens posterodorsally and is about 1.7 mm in diameter. The angular process is incompletely preserved, but seems to have been narrow (c. 68[0]) and did not form a medial shelf (c. 69[0]), similar to the condition in *P. starri* (LACM 115153).

The postcanine dentition of LACM 159981 is similar to the holotype, only differing slightly in dimensions (Table 2) and as in OCPC 5710 it is missing p1 and m2. The alveoli for the incisors are not preserved. The canine is conical and recurved posterodorsally. Medially, the canine has a longitudinal, medially concave wear facet likely caused by friction with the lateral-most upper incisor. The alveoli for p1 and m2 are both for single-rooted teeth, and there is no indication that the m2 root was bilobed as in *Eotaria citrica*.

## Discussion

### Sexual dimorphism

As in many other carnivores, most extant pinnipeds exhibit some degree of sexual dimorphism (Ralls & Mesnick, 2002). In some fossil taxa, sexual dimorphism has been proposed as well, including desmatophocids, the stem pinnipeds *Enaliarctos emlongi* Berta, 1991, *Pteronarctos goedertae* Barnes, 1989, and the otariid *Thalassoleon mexicanus*, amongst others (Mitchell, 1966; Berta, 1994; Deméré & Berta, 2002, 2005; Cullen et al., 2014). If sexual dimorphism was indeed present in *E. emlongi* and *P. goedertae*, then the implication is that this characteristic is ancestral to pinnipeds in general, and thus, it would be expected to be present throughout their evolutionary history. However, as mentioned by Cullen et al. (2014), some aspects of sexual dimorphism, such as size variation, seem to be more complex and may have been lost and regained in some groups (e.g., phocids) or evolved iteratively (e.g., desmatophocids). Because of this, it may be best to explore aspects of sexual dimorphism on a group-by-group basis.

Nevertheless, considering the possibility that sexual dimorphism is ancestral within pinnipeds, additional comparisons of *Eotaria crypta* and *Eotaria citrica* with crown otariids were made. These included investigating whether differences in position of the genial tuberosity, robustness of the mandible and overall dimensions of the mandible of both species of *Eotaria* are the result of intraspecific variation and/or sexual dimorphism. These features were examined in *P. starri* (*n* = 9), *Callorhinus ursinus* (*n* = 10), *E. jubatus* (*n* = 11) and *Z. californianus* (*n* = 70) (Table S1). The observations show that in *P. starri*, the genial tuberosity was consistently located below p3 (e.g., Fig. 6B; Table S1). Meanwhile, in *E. jubatus* it was located below p2 or the gap between p1 and p2 in nearly all of the specimens examined (Table S1). The only two exceptions being a young male (LACM 620) and another young individual of undetermined sex that was likely a male (LACM 51173), in which the tuberosity was positioned more posteriorly. In the sample of *Callorhinus ursinus* the tuberosity was uniformly located below the posterior half of p2, while in *Z. californianus* it was located under the gap between p2 and p3 in all, but one of the adult males (LACM 31360), which had the tuberosity located more anteriorly, between p1 and p2 (Table S1). These observations suggest that intraspecific variation in the position of the genial tuberosity is negligible and cannot account for the differences in this feature between both species of *Eotaria*.

The difference in robustness of the mandible has been used qualitatively as a characteristic that sets apart male and female morphs of some Pliocene otariids (e.g., *Callorhinus gilmorei*; Berta & Deméré, 1986; Boessenecker, 2011). Here, this character was explored quantitatively by comparing the ratio between width and height of the horizontal ramus at p4 (Table S1). The measurements of the extant taxa sampled show that males of *Z. californianus*, *Callorhinus ursinus* and *E. jubatus* have ratios that are between 13 and 15% higher than their female counterparts. On the other hand, the difference between both species of *Eotaria* is much greater (22%) than what was expected based on the extant taxa (Table S1). The phylogenetic significance of this character needs to be explored further, but can be, at present, interpreted as an additional feature separating both species of *Eotaria*.

**Figure 4:**
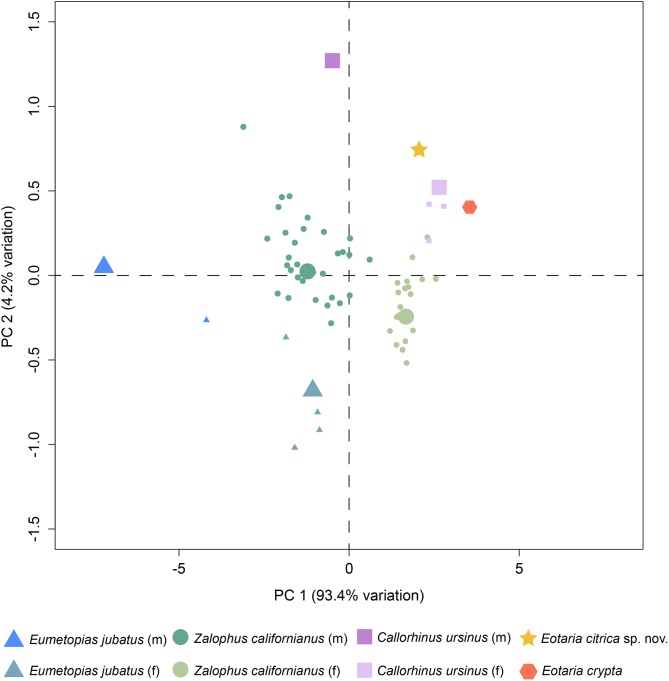
Principal component analysis of pan-otariid mandibles. Analysis based on mandibular measurements of adult specimens of known sex. Male and female morphs are color and shape coded. PCA1 represents most of the variance of the sample. Larger shapes represent the medoids (computed as the specimen of the dataset whose average dissimilarity to all of the objects in the sex-cluster is minimal).

Finally, a preliminary PCA was performed comparing a set of mandible measurements in order to test for sexual dimorphism. The results of the analysis show that PC1 explains 93.4% of the variance and represents diameter of canine and height of ramus. Meanwhile, PC2 represents 4.2% of the variance and represents total length of the mandible and width of the ramus (Table S2). Combined, PC1 and PC2 explain 97.6% of the total variability. In agreement with the sexual dimorphism widely proposed for otariids, there is a clear separation of female and male groups, with females having more positive values along the PC1, relative to males and both *Eotaria citrica* and *Eotaria crypta* are placed in the positive (female) side of the PC1 (Fig. 4). Furthermore, the degree of sexual dimorphism among species was assessed, and compared with the morphological differences between *Eotaria citrica* and *Eotaria crypta.* For this, the medoids of female and male groups (represented as the larger shapes in Fig. 4), and the distance between medoids, were calculated and the distance values between sexes of each species were compared with the distance between *Eotaria citrica* and *Eotaria crypta*. The medoids, rather than the centroids, were preferred given the small sample size of each sex of some species and because this measure includes members of the data sets (Struyf, Hubert & Rousseeuw, 1997). These numbers show that the morphological distance between *Eotaria citrica* and *Eotaria crypta* is about half the distance between females and males of *Z. californianus* and *Callorhinus ursinus,* and about one quarter the distance between females and males of *E. jubatus* (Table S3). Together, the quantitative analyses using the morphological dimensions of the species studied suggest that although similar, *Eotaria citrica* and *Eotaria crypta* do not correspond to female and male representatives of the same species. However, because of the limited sampling, these results could be interpreted differently. Alternatively, the close distance between species of *Eotaria* could mean that there was less sexual dimorphism in stem otariids, in contrast to extant taxa (e.g., *Callorhinus ursinus*). Nevertheless, differentiation between both species of *Eotaria* is still supported by the diagnostic characters described earlier in this work. Future analyses with larger samples of *Eotaria* spp., *Callorhinus ursinus*, as well as *P. starri* are needed to provide better insight into early pan-otariid sexual dimorphism.

### Relationships and comparison

**Figure 5:**
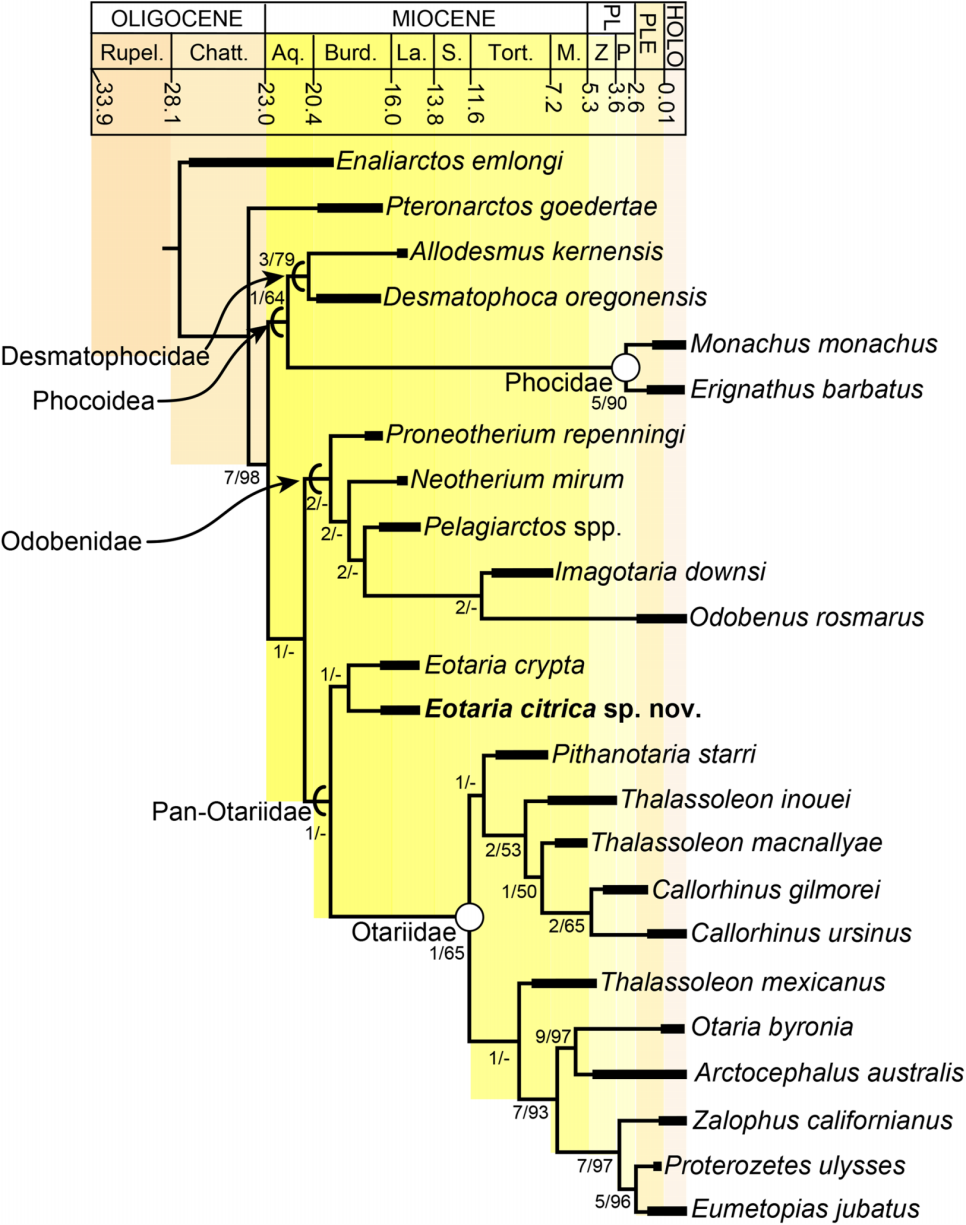
Time calibrated phylogeny of pan-otariidae. Phylogenetic tree based on most parsimonious tree of length = 451, CI = 0.430, RI = 0.559. Arcs denote stem-based taxa, while closed circles denote node-based clades; number at nodes indicate decay indices/bootstrap values. Abbreviations: Aq., Aquitanian; Burd., Burdigalian; Chatt., Chattian; La., Langhian; M., Messinian; P, Piacenzian; PL, Pliocene; PLE, Pleistocene; Rupel., Rupelian; S. Serravalian; Tort., Tortonian; Z, Zanclean. Age ranges of species based on Boessenecker & Churchill (2015); time scale based on Cohen et al. (2013).

The phylogenetic analysis resulted in a single most parsimonious tree of length = 451, consistency index = 0.430 and retention index = 0.559 (Fig. 5). The resulting topology is in agreement with that of Boessenecker & Churchill (2015: Fig. 2) with respect to the position of most taxa. Furthermore, the results of the phylogenetic analysis are used here as the basis for a phylogenetic definition of the group following the examples provided by Joyce, Parham & Gauthier (2004) and guidelines from Cantino & de Queiroz (2014). In addition to defining Pan-Otariidae as the group comprising stem and crown taxa (see above), Otariidae Gill, 1866, is phylogenetically defined as the crown group composed of the last common ancestor of *Callorhinus ursinus*, the *Eumetopias* + *Zalophus* clade (= “Northern sea lion clade” of Churchill, Boessenecker & Clementz, 2014), the *Otaria* + *Arctocephalus* clade (= “Southern otariid clade” of Churchill, Boessenecker & Clementz, 2014) and all its descendants.

**Figure 6:**
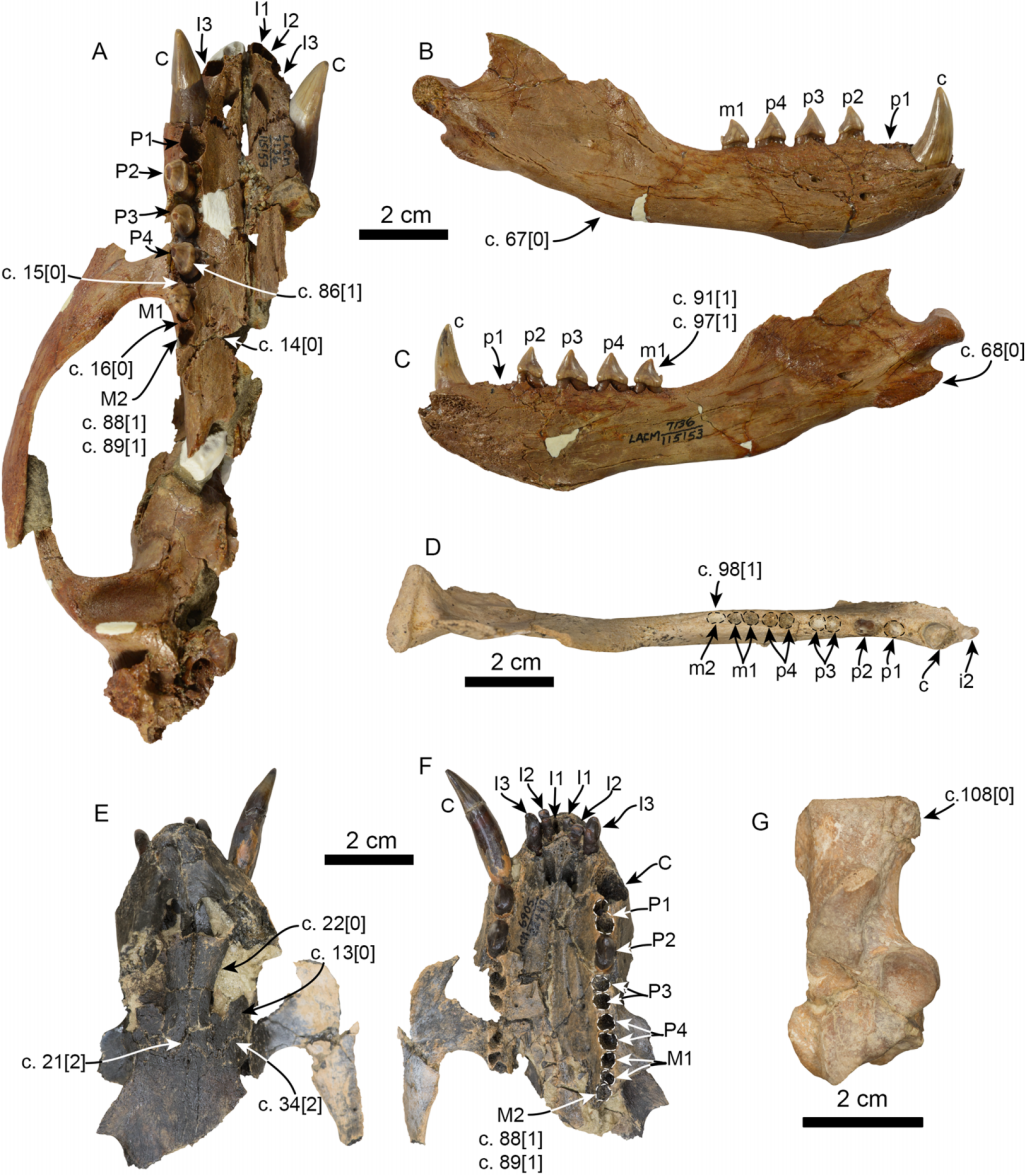
Additional specimens of *Pithanotaria starri* used in the phylogenetic analysis. LACM 115153, partial skull in ventral view (A), right mandible in lateral (B) and medial (C) views. LACM 115677, right mandible in occlusal view (D). LACM 22449, partial skull in dorsal (E) and ventral (F) views. LACM 52773, right calcaneus in astragalar view (G). Newly scored or modified characters are indicated arrows and the respective scoring for each state. Abbreviations: c., character state as described and numbered by Boessenecker & Churchill (2015), e.g. (c. 15[0]) refers to state 0 of character 15.

In the phylogenetic analysis, *Eotaria* spp. occupies the basalmost position amongst pan-otariids, sharing with later otariids the reduction of the metaconid and lanceolate shape of the protoconid (c. 94[3]). In general, the morphologies of both species display a number of characteristics that are reminiscent of more basal pinnipeds. One of these is the retention of m2, a plesiomorphic characteristic that amongst otariids is variably present in *P. starri* (Fig. 6D), but normally absent in all other known otariids. However, in *Eotaria crypta* the root of m2 is rounded, while in *Eotaria citrica* it is bilobed, resembling the condition in *E. emlongi* (Berta, 1991). Both “Topanga” taxa also display a very reduced metaconid, while otariids are characterized by the complete loss of this cusp (e.g., *P. starri*, Figs. 6B and 6C). *Eotaria citrica* differs further from *Eotaria crypta* by the more posterior position of the genial tuberosity, p1 with a proportionately smaller root, p2–4 with partially crenulated lingual cingula, as well as its larger size and proportionately more robust horizontal ramus (similar to some specimens of *Callorhinus gilmorei*; LACM 115253). Furthermore, as discussed above, intraspecific variation and sexual dimorphism does not seem to account for the differences between these two taxa. However, it should be noted that even though the resulting topology of the phylogenetic analysis shows both species as sister taxa (Fig. 5), support for that clade is low (bootstrap <50%), and the clade would collapse in trees that are one step longer. Future discoveries of additional material referable to *Eotaria crypta* and *Eotaria citrica* may clarify further the relationship amongst these two species.

Currently *Eotaria citrica* and *Eotaria crypta* represent the oldest known pan-otariids, which, as discussed by Boessenecker & Churchill (2015), shows that the group evolved in the North Pacific Ocean. Interestingly, both taxa have yet to be recovered from the nearly contemporaneous, densely fossiliferous and well-sampled Sharktooth Hill bonebed of the Round Mountain Silt (Pyenson et al., 2009). All other known Sharktooth Hill pinnipeds are known from the “Topanga” Fm. as well (Howard & Barnes, 1987; Boessenecker & Churchill, 2013; Garibay, Velez-Juarbe & Parham, 2016). However, the similarities seem to be limited to the generic level. Studies of *Pelagiarctos* and *Allodesmus* from the “Topanga” Fm. hint at species-level taxonomic differences from their Sharktooth Hill counterparts, indicating some differences between the pinniped faunas of these formations (Boessenecker & Churchill, 2013; Garibay, Velez-Juarbe & Parham, 2016). These differences aside, it is hypothesized that *Eotaria* or some other pan-otariid should occur in the Sharktooth Hill fauna. Their absence may be taphonomic or due to differential preferences and availability of coastal habitats, as is the case with some extant sympatric pinnipeds (e.g., Arias-del-Razo et al., 2016) or that early otariids were more pelagic, as suggested by Kohno (2004).

### Pinniped faunas of Southern California

**Figure 7:**
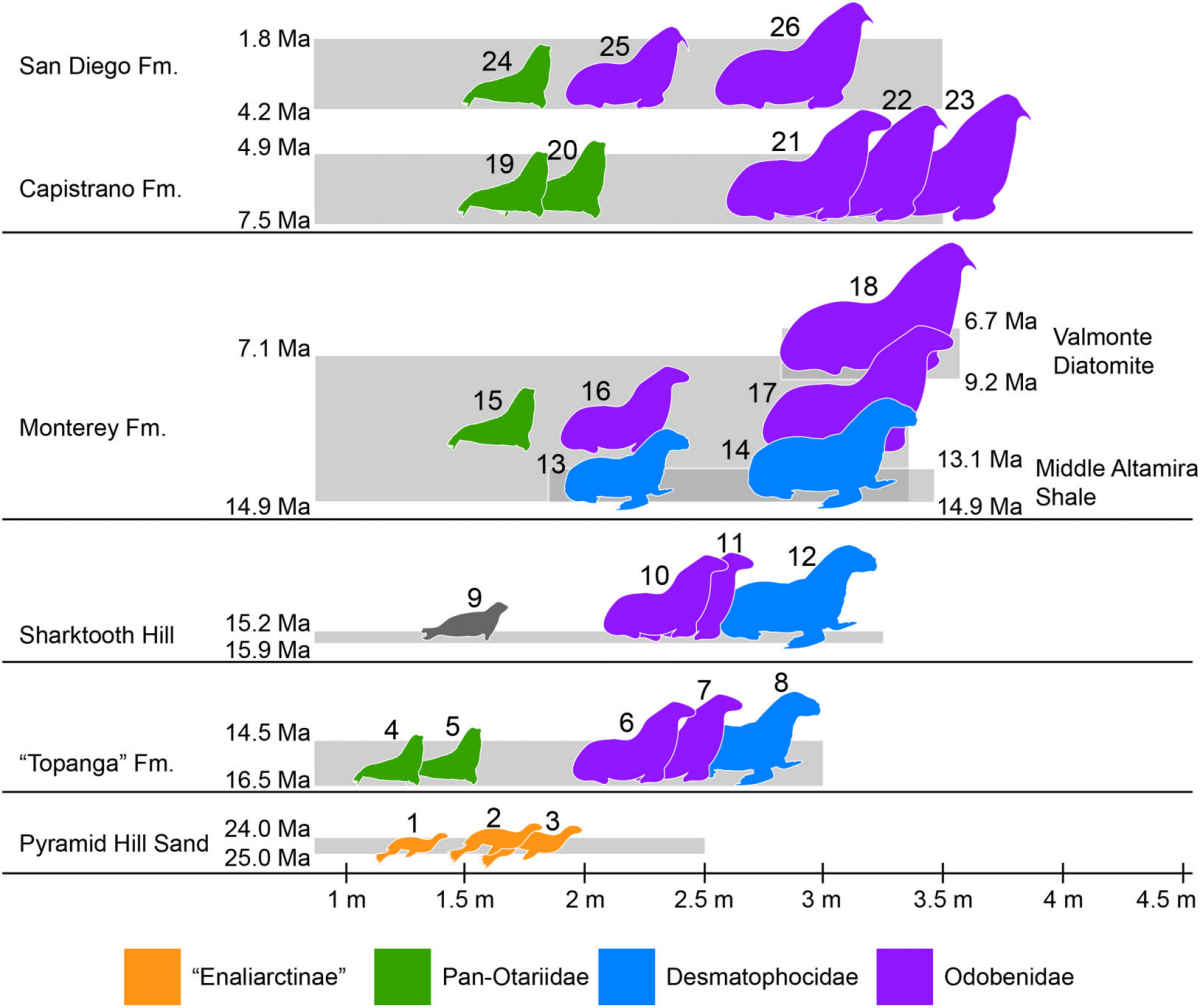
Representative pinniped faunas of Southern California. Pinniped faunas range from Oligocene to early Pliocene (25–4.9 Ma) and are based on published and examined museum records. (1) *Pinnarctidion bishopi* Barnes, 1979; (2) *Enaliarctos mitchelli* Barnes, 1979; (3) *Enaliarctos mealsi* Mitchell & Tedford, 1973; (4) *Eotaria crypta*; (5) *Eotaria citrica*; (6) *Neotherium* sp.; (7) *Pelagiarctos* sp.; (8) *Allodesmus* sp.; (9) pinnipedia indet.; (10) *Neotheirum mirum*; (11) *Pelagiarctos thomasi*; (12) *Allodesmus kelloggi*; (13) *Atopotarus courseni*; (14) *Allodesmus* sp.; (15) *Pithanotaria starri*; (16) Odobenidae sp. 1; (17) Odobenidae sp. 2; (18) Odobenidae sp. 3; (19) Otariidae sp. 1; (20) *Thalassoleon* sp.; (21) Odobenidae sp. 4; (22) Odobenidae sp. 5; (23) *Gomphotaria pugnax*; (24) *Callorhinus gilmorei*; (25) *Valenictus chulavistensis*; (26) *Dusignathus seftoni*. For the body lengths see Table S4. Silhouettes are based on Mitchell (1966), Berta & Ray (1990), Reeves, Stewart & Leatherwood (1992), Shirihai & Jarret (2006) and Steven Traver through phylopic.org.

Southern California is known for its exceptional record of fossil marine mammals that provides a unique perspective of the evolutionary history of different groups over the last ∼30 million years (e.g., Barnes, 1976; Domning, 1978). A preliminary specimen-based overview of the pinniped assemblages from Southern California show a nearly continuous record spanning from around 25 to 2 million years ago (Fig. 7; Table S4). The oldest pinnipeds in the region are stem taxa known from the Chattian Pyramid Hill Sand (25–24 Ma; Hosford Scheirer & Magoon, 2007). All three have relatively small body sizes (<2 m long; Churchill, Clementz & Kohno, 2015; Fig. 7), which is considered to be the plesiomorphic condition for the group. By the upper Burdigalian through lower Langhian (between 16.5 and 14.5 Ma) these stem pinnipeds had apparently become locally extinct. This time also marks the appearance of the earliest crown pinnipeds in the “Topanga” Formation (16.5–14.5 Ma). As discussed earlier, the “Topanga” and the nearly contemporaneous (15.9–15.2 Ma; Pyenson et al., 2009) Sharktooth Hill faunas are characterized by the occurrence of two odobenids, *Neotherium* and *Pelagiarctos*, as well as the desmatophocid *Allodesmus*. The taxon that seems to be conspicuously missing from Sharktooth Hill is the pan-otariid *Eotaria*, although there are fragmentary remains (e.g., LACM 127710) that seem to hint at the presence of a small pinniped of uncertain affinities that could be this taxon. In both assemblages, *Allodesmus* has the largest body size, exceeding 2.5 m in length, while the odobenids were intermediate in size (2–2.5 m) and otariids the smallest (>2 m) (Fig. 7; Table S4).

At least two more species of *Allodesmus* are known from deposits younger than Sharktooth Hill and the “Topanga” Fm. in Southern California. *Atopotarus courseni* Downs, 1956, and *Allodesmus* sp. (represented by LACM 160016), both from the Langhian middle Altamira Shale (14.9–13.1 Ma; Blake, 1991; Barron & Isaacs, 2001), represent the chronologically youngest well-constrained record of *Allodesmus* in the region. Furthermore, LACM 160016 represents the largest body size estimate in any species of *Allodesmus*, exceeding 3 m in length (Fig. 7; Table S4). Barnes (1978) reported the occurrence of *Allodesmus* in late Miocene deposits of the Monterey Fm. in Orange County, CA, USA. However, no specific details regarding the age assigned to that locality were given and the Monterey Fm. in this area ranges from mid Langhian through upper Tortonian (14.9–7.1 Ma; Smith, 1960; Blake, 1991; Barron & Isaacs, 2001). So it remains uncertain whether this represents a late Miocene or older record of *Allodesmus,* coeval and/or conspecific with those of the Altamira Shale. Additional remains from the Monterey Fm. that were originally thought to represent *Allodesmus* are now known to be those of an odobenid (Downs, 1955; Barnes, 1985). Other pinnipeds from the Monterey Fm. include what potentially is the oldest crown otariid *P. starri* as well as two odobenids (Fig. 7; Table S4). Regardless of whether *Allodesmus* was coeval with these other pinnipeds, odobenids represent the intermediate (2–2.5 m) and large (>2.5 m) body size taxa within the Monterey fauna, while otariids were the smallest (<2 m). The upper part of the Monterey Fm. partially correlates with the mid Tortonian to early Messinian Valmonte Diatomite (9.2–6.7 Ma; Blake, 1991; Barron & Isaacs, 2001). The only pinniped known from this formation is a large (>3 m) odobenid, variously identified as *Pontolis magnus* True, 1905, (Lyon, 1941) or as a species similar to *Imagotaria downsi* Mitchell, 1968, by seemingly representing a new undescribed taxon (JVJ, 2016, personal observation). The general pattern from the mid Langhian through early Messinian (14.9–6.7 Ma) shows the local extinction of desmatophocids and a shift in the body size pattern seen in earlier deposits where *Allodesmus* was the largest pinniped in the faunas (Fig. 7; Churchill, Clementz & Kohno, 2015).

The end of the Miocene and beginning of the Pliocene is represented by the upper Messinian to lower Zanclean Capistrano Formation (Deméré & Berta, 2005; Barboza et al., in press). By this time there is nearly a two-fold increase in the maximum body size of pinnipeds, exemplified by the large odobenids known from this formation (Fig. 7). Otariids are present in the Capistrano Fm. as well, but as in earlier deposits, they had the smallest body size in the fauna (∼2 m). The Capistrano pinniped assemblage includes several odobenids, including *G. pugnax*. This particular species displays morphological features that are indicative that it was a benthic feeder (Barnes & Raschke, 1991), and may represent the first occurrence of this feeding mode in odobenids. Lastly, the youngest of the formations used in this study is the Zanclean to Gelasian San Diego formation (Wagner et al., 2001; Vendrasco et al., 2012). Three pinniped taxa are known from this formation, the crown otariid *Callorhinus gilmorei* and the odobenids *Valenictus chulavistensis* Deméré, 1994, and *Dusignathus seftoni* Deméré, 1994 (Fig. 7). The San Diego fauna could be considered as transitional between the Capistrano and late Pleistocene/recent assemblages from California. This is mainly due to the occurrence of *Callorhinus*, a taxon nowadays represented in the region by *Callorhinus ursinus* and of *V. chulavistensis*, a taxon morphological similar to *O. rosmarus* which once inhabited Northern California (Harington, 2008).

The overall pattern of body size increase of pinnipeds in the region (Fig. 7; Table S4; Churchill, Clementz & Kohno, 2015) is similar to what is observed in other marine mammals (Pyenson & Vermeij, 2016), as well as in shallow-water mollusks (Vermeij, 2012). In addition, there is a notable shift in which taxa are the largest in the assemblages. In earlier deposits (i.e. “Topanga,” Sharktooth Hill and middle Altamira) the desmatophocid *Allodesmus* was the largest taxon, but by the mid Langhian through upper Tortonian (14.9–7.1 Ma; Monterey fauna), *Allodesmus* became extinct and odobenids diversified and became the mid to large pinnipeds in the faunas (7.5–4.9 Ma; Capistrano fauna). Similarly, during this period (mid Miocene-early Pliocene), there is a notable change in the marine bird faunas of California (Kloess & Parham, 2017). The observed increase in body sizes (pinnipeds, cetaceans and mollusks), faunal changes (pinnipeds, marine birds) and odobenid diversification were likely the result of increasing marine productivity in the Neogene of the North Pacific (Vermeij, 2011; Churchill, Clementz & Kohno, 2014; Kloess & Parham, 2017).

Odobenid maximum body size seems to decrease in post-Capistrano deposits (Fig. 7). Nevertheless, they continued to be the largest pinnipeds within the faunas throughout the Pliocene/Pleistocene San Diego Fm., up until the appearance of the phocid *Mirounga angustirostris* in the region later in the Pleistocene (Miller, 1971; Churchill, Clementz & Kohno, 2015). This is evidently part of the marine vertebrate faunal turnover that occurred in the Eastern Pacific Region at or near the Pliocene–Pleistocene boundary, which may have been a result of global or local oceanographic changes (Boessenecker, 2013; Valenzuela-Toro et al., 2013, 2016).

Ultimately, further work aimed at more precise dates for the Monterey Fm. localities in Southern California is needed to properly establish the timing of the extinction of *Allodesmus* and the middle to late Miocene pinniped fauna turnover, as well as the earliest records of *Pithanotaria* in this region. This, combined with more precise estimates of feeding preferences in extinct pinnipeds using craniomandibular and dental proxies (e.g., Kelley & Motani, 2015; Churchill & Clementz, 2015), is needed in order to reach a more detailed understanding of the paleoecology of these extinct pinniped faunas.

## Conclusion

The stem otariid *Eotaria citrica* sp. nov., was described from the upper Burdigalian to lower Langhian “Topanga” Formation in Orange County, CA, USA. The new species shows a combination of characters shared with the coeval *Eotaria crypta*, also from the “Topanga” Fm. However, both species differ in phylogenetically significant mandibular and dental characters that cannot be justified by sexual and/or intraspecific variation. Altogether, these two species represent the earliest pan-otariids, further supporting the hypothesis that this group of marine mammals originated in the North Pacific Region. The discovery of *Eotaria citrica* elevates the number of pinniped taxa in the “Topanga” to a total of five, including *Eotaria crypta*, a desmatophocid and two odobenids, some which represent the oldest records of crown pinnipeds in California. A comparison across the different pinniped faunas in Southern California showed that the “Topanga” is most similar to the Sharktooth Hill bonebed fauna, with the exception that *Eotaria* seems to be absent in the latter.

Additionally, a preliminary overview of the late Oligocene through early Pleistocene pinniped faunas of Southern California shows an overall increase in body size over time. During the middle Miocene the largest pinniped in the faunas were desmatophocids, while odobenids occupied a mid-size range and otariids were the smallest. After the extinction of desmatophocids around the early late Miocene, odobenids diversified and occupied the mid and large body size ranges from around the late Miocene through early Pleistocene, usually approaching or exceeding 3 m in body length, while otariids continued to have the smallest body sizes in the assemblages, barely exceeding 2 m in length. This pattern differs in part from modern assemblages where the phocid *M. angustirostris* is the largest pinniped in the assemblages, odobenids are extinct and the mid to small size ranges are occupied by otariids (e.g., *Z. californianus*) or phocids (e.g., *P. vitulina*).

## Supplemental Information

10.7717/peerj.3022/supp-1 Supplemental Information 1 Character/state matrix used for phylogenetic analysis.

10.7717/peerj.3022/supp-2 Supplemental Information 2 Table S1. Measurements (in mm) and morphological features observed for sexual dimorphism and intraspecific variation.

10.7717/peerj.3022/supp-3 Supplemental Information 3 Table S2. Loadings of PC1 and PC2.

10.7717/peerj.3022/supp-4 Supplemental Information 4 Table S3. Distance between medoids of each cluster of species included in the principal component analysis.

10.7717/peerj.3022/supp-5 Supplemental Information 5 Table S4. List of specimens used for body length estimates and their corresponding lengths based on equations from Churchill, Clementz & Kohno (2015).

## Institutional Abbreviations

c.: Character state as described and numbered by Boessenecker & Churchill (2015), e.g. (c. 74[0]) refers to state 0 of character 74
CBMPV: Natural History Museum and Institute, Chiba, Department of Earth Sciences, Vertebrate Paleontology Collection
IGCU: Instituto de Geología, Ciudad Universitaria, Universidad Nacional Autónoma de México
LACM: Vertebrate Paleontology Collection, Natural History Museum of Los Angeles County, Los Angeles, CA, USA
LACM Loc.: Vertebrate Paleontology Locality, Natural History Museum of Los Angeles County, Los Angeles, CA, USA
LC: Ralph B. Clark (formerly “Los Coyotes”) Regional Park, Interpretive Center, Buena Park, CA, USA
OCPC: Orange County Paleontological Collection, John D. Cooper Archaeological and Paleontological Center, Santa Ana, CA, USA
SDSNH: San Diego Natural History Museum, San Diego, CA, USA.

## Additional Information and Declarations

### Data Deposition

The following information was supplied regarding data availability: The raw data has been supplied as Supplemental Dataset Files.

### New Species Registration

The following information was supplied regarding the registration of a newly described species: Eotaria citrica Taxon LSID: urn:lsid:zoobank.org:act:BC519C1F-6E1C-4BDB-81CB-D67C18090A4A. Publication LSID: urn:lsid:zoobank.org:pub:AA9D04DC-A79E-49AC-85AE-744D7BA39D6D.

## References

[ref-1] Aranda-Manteca FJ, Domning DP, Barnes LG (1994). A new middle Miocene sirenian of the genus *Metaxytherium* from Baja California and California: relationships and paleobiogeographic implications. Proceedings of the San Diego Society of Natural History.

[ref-2] Arias-del-Razo A, Heckel G, Schramm Y, Pardo MA (2016). Terrestrial habitat preferences and segregation of four pinniped species on the islands off the western coast of the Baja California Peninsula, Mexico. Marine Mammal Science.

[ref-3] Barboza MM, Parham JF, Santos G-P, Kussman BN, Velez-Juarbe J (in press). The age of the Oso member, Capistrano formation, and a review of fossil crocodylians from California. Paleobios.

[ref-4] Barnes LG (1976). Outline of eastern North Pacific fossil cetacean assemblages. Systematic Zoology.

[ref-5] Barnes LG (1978). A review of *Lophocetus* and *Liolithax* and their relationships to the delphinoid family Kentriodontidae (Cetacea: Odontoceti). Natural History Museum of Los Angeles Science Bulletin.

[ref-6] Barnes LG (1979). Fossil enaliarctine pinnipeds (Mammalia: Otariidae) from Pyramid Hill, Kern County, California. Contributions in Science.

[ref-7] Barnes LG (1985). The late Miocene dolphin *Pithanodelphis* Abel, 1905 (Cetacea: Kentriodontidae) from California. Contributions in Science.

[ref-8] Barnes LG (1988). A new fossil pinniped (Mammalia: Otariidae) from the middle Miocene Sharktooth Hill Bonebed, California. Contributions in Science.

[ref-9] Barnes LG (1989). A new enaliarctine pinniped from the Astoria formation, Oregon and a classification of the Otariidae (Mammalia: Carnivora). Contributions in Science.

[ref-10] Barnes LG, Raschke RE (1991). *Gomphotaria pugnax*, a new genus and species of late Miocene dusignathine otariid pinniped (Mammalia: Carnivora) from California. Contributions in Science.

[ref-77] Barnes LG, Ray CE, Koretsky IA, Csiiki Z (2006). A new Pliocene sea lion, *Proterozetes ulysses* (Mammalia: Otariidae) from Oregon, U.S.A. Mesozoic and Cenozoic Vertebrates and Paleoenvironments: Tributes to the Career of Prof. Dan Grigorescu.

[ref-11] Barron JA, Isaacs CM, Isaacs CM, Rullkotter J (2001). Updated chronostratigraphic framework for the California Miocene. The Monterey Formation–From Rocks to Molecules.

[ref-12] Berta A (1991). New *Enaliarctos** (Pinnipedimorpha) from the Oligocene and Miocene of Oregon and the role of “Enaliarctids” in pinniped phylogeny. Smithsonian Contributions to Paleobiology.

[ref-13] Berta A (1994). New specimens of the pinnipediform *Pteronarctos* from the Miocene of Oregon. Smithsonian Contributions to Paleobiology.

[ref-14] Berta A, Deméré TA (1986). *Callorhinus gilmorei* n. sp. (Carnivora: Otariidae) from the San Diego Formation (Blancan) and its implications for otariid phylogeny. Transactions of the San Diego Society of Natural History.

[ref-15] Berta A, Ray CE (1990). Skeletal morphology and locomotor capabilities of the archaic pinniped *Enaliarctos mealsi*. Journal of Vertebrate Paleontology.

[ref-16] Berta A, Sumich JL, Kovacs KM (2015). Marine Mammals: Evolutionary Biology.

[ref-17] Blake GH (1991). Review of the Neogene biostratigraphy and stratigraphy of the Los Angeles basin and implications for basin evolution. AAPG Memoir.

[ref-18] Boessenecker RW (2011). New records of the fur seal *Callorhinus* (Carnivora: Otariidae) from the Plio-Pleistocene Rio Dell Formation of Northern California and comments on otariid dental evolution. Journal of Vertebrate Paleontology.

[ref-19] Boessenecker RW (2013). A new marine vertebrate assemblage from the late Neogene Purisima Formation in Central California, part II: pinnipeds and cetaceans. Geodiversitas.

[ref-20] Boessenecker RW, Churchill M (2013). A reevaluation of the morphology, paleoecology, and phylogenetic relationships of the enigmatic walrus *Pelagiarctos*. PLoS ONE.

[ref-21] Boessenecker RW, Churchill M (2015). The oldest known fur seal. Biology Letters.

[ref-22] Bowdich TE (1821). An Analysis of the Natural Classification of Mammalia, for the Use of Students and Travelers.

[ref-23] Cantino PD, de Queiroz K (2014). http://www.ohiou.edu/phylocode.

[ref-24] Churchill M, Clementz MT (2015). Functional implications of variation in tooth spacing and crown size in Pinnipedimorpha (Mammalia: Carnivora). Anatomical Record.

[ref-25] Churchill M, Boessenecker RW, Clementz MT (2014). Colonization of the Southern Hemisphere by fur seals and sea lions (Carnivora: Otariidae) revealed by combined evidence phylogenetic and Bayesian biogeographical analysis. Zoological Journal of the Linnean Society.

[ref-26] Churchill M, Clementz MT, Kohno N (2014). Predictive equations for the estimation of body size in seals and sea lions (Carnivora: Pinnipedia). Journal of Anatomy.

[ref-27] Churchill M, Clementz MT, Kohno N (2015). Cope’s rule and the evolution of body size in Pinnipedimorpha (Mammalia: Carnivora). Evolution.

[ref-28] Cohen KM, Finney SC, Gibbard PL, Fan JX (2013). The ICS international chronostratigraphic chart. Episodes.

[ref-29] Cullen TM, Fraser D, Rybczynski N, Schröder-Adams C (2014). Early evolution of sexual dimorphism and polygyny in Pinnipedia. Evolution.

[ref-30] Deméré TA (1994). Two new species of fossil walruses (Pinnipedia: Odobenidae) from the upper Pliocene San Diego Formation, California. Proceedings of the San Diego Society of Natural History.

[ref-31] Deméré TA, Berta A (2002). The Miocene pinniped *Desmatophoca oregonensis* Condon, 1906 (Mammalia: Carnivora), from the Astoria Formation, Oregon. Smithsonian Contributions to Paleobiology.

[ref-32] Deméré TA, Berta A (2005). New skeletal material of *Thalassoleon* (Otariidae: Pinnipedia) from the late Miocene-early Pliocene (Hemphillian) of California. Bulletin of the Florida Museum of Natural History.

[ref-33] Domning DP (1978). Sirenian evolution in the North Pacific Ocean. University of California Publications in Geological Sciences.

[ref-34] Downs T (1955). A fossil sea lion from the Miocene of the San Joaquin Hills, Orange County, California. Bulletin of the Southern California Academy of Sciences.

[ref-35] Downs T (1956). A new pinniped from the Miocene of Southern California: with remarks on the Otariidae. Journal of Paleontology.

[ref-36] Garibay A, Velez-Juarbe J, Parham JF (2016). New material of the stem seal *Allodesmus* from the Topanga Formation of Orange County. GSA Cordilleran Section Abstract with Programs.

[ref-37] Gill T (1866). Prodrome of a monograph of the pinnipedes. Proceedings of the Essex Institute.

[ref-38] Harington CR (2008). The evolution of arctic marine mammals. Ecological Applications.

[ref-39] Hosford Scheirer AH, Magoon LB (2007). Age, distribution, and stratigraphic relationship of rock units in the San Joaquin Basin province, California. U.S. Geological Survey Professional Paper.

[ref-40] Howard H, Barnes LG (1987). Middle Miocene marine birds from the foothills of the Santa Ana Mountains, Orange County, California. Contributions in Science.

[ref-41] Illiger JKW (1811). Prodromus Systematis Mammalium et Avium Additis Terminis Zoographicis Utriusque Classis, Eorumque Versione Germanica.

[ref-42] Joyce WC, Parham JF, Gauthier J (2004). Developing a protocol for the conversion of rank-based taxon names to phylogenetically defined clade names, as exemplified by turtles. Journal of Paleontology.

[ref-43] Kelley NP, Motani R (2015). Trophic convergence drives morphological convergence in marine tetrapods. Biology Letters.

[ref-44] Kellogg R (1922). Pinnipeds from Miocene and Pleistocene deposits of California. University of California Publications, Bulletin of the Department of Geological Sciences.

[ref-45] Kellogg R (1925). New pinnipeds from the Miocene diatomaceous earth near Lompoc, California. Contributions to Paleontology from the Carnegie Institution of Washington.

[ref-46] Kellogg R (1931). Pelagic mammals from the Temblor Formation of the Kern River region, California. Proceedings of the California Academy of Sciences.

[ref-47] Kloess PA, Parham JF (2017). A specimen-based approach to reconstructing the late Neogene seabird communities of California. Palaeogeography, Palaeoclimatology, Palaeoecology.

[ref-48] Kohno N (1992). A new Pliocene fur seal (Carnivora: Otariidae) from the Senhata Formation on the Boso Peninsula, Japan. Natural History Research.

[ref-49] Kohno N (2004). Ecological shift in the otariid pinnipeds from pelagic to inshore: evidence from the Middle Miocene record of the North Pacific. Journal of Vertebrate Paleontology.

[ref-50] Lesson RP, Bory de Saint Vincent BGM (1828). Histoire naturelle des Phoques. Dictionnaire classique d’histoire naturelle.

[ref-51] Linnaeus C (1758). Systema Naturae per Regna tria Naturae, Secundum Classes, Ordines, Genera, Species, cum Characteribus, Differentiis, Synonymis, Locis.

[ref-52] Lyon GM (1941). A Miocene sea lion from Lomita, California. University of California Publications in Zoology.

[ref-53] Miller WE (1971). Pleistocene vertebrates of the Los Angeles Basin and vicinity (exclusive of Rancho La Brea). Bulletin of the Los Angeles County Museum of Natural History.

[ref-54] Mitchell ED (1966). The Miocene pinniped *Allodesmus*. University of California Publications in Geological Sciences.

[ref-78] Mitchell ED (1968). The Mio-Pliocene pinniped *Imagotaria*. Journal of the Fisheries Research Board of Canada.

[ref-55] Mitchell ED, Tedford RH (1973). The Enaliarctinae. A new group of extinct aquatic Carnivora and a consideration of the origin of the Otariidae. Bulletin of the American Museum of Natural History.

[ref-56] Ogg JG, Ogg G, Gradstein FM (2008). The Concise Geologic Time Scale.

[ref-57] Pyenson ND, Vermeij GJ (2016). The rise of ocean giants: maximum body size in Cenozoic marine mammals as an indicator for productivity in the Pacific and Atlantic Oceans. Biology Letters.

[ref-58] Pyenson ND, Irmis RB, Lipps JH, Barnes LG, Mitchell ED, McLeod SA (2009). Origin of a widespread marine bonebed deposited during the middle Miocene Climatic Optimum. Geology.

[ref-59] R Development Core Team (2012). R: a language and environment for statistical computing.

[ref-60] Ralls K, Mesnick SL, Perrin WF, Würsig B, Thewissen JGM (2002). Sexual dimorphism. Encyclopedia of Marine Mammals.

[ref-61] Raschke RE (1984). Early and middle Miocene vertebrates from the Santa Ana Mountains, California. Memoirs of the Natural History Foundation of Orange County.

[ref-62] Reeves RR, Stewart BS, Leatherwood S (1992). The Sierra Club Handbook of Seals and Sirenians.

[ref-63] Repenning CA, Tedford RH (1977). Otarioid seals of the Neogene. U.S. Geological Survey Professional Paper.

[ref-64] Shirihai H, Jarret B (2006). Whales, Dolphins and Seals: A Guide to the Marine Mammals of the World.

[ref-65] Smith PB (1960). Foraminifera of the Monterey Shale and Puente Formation, Santa Ana Mountains and San Juan Capistrano Area, California. U.S. Geological Survey Professional Paper.

[ref-66] Struyf A, Hubert M, Rousseeuw P (1997). Clustering in an object-oriented environment. Journal of Statistical Software.

[ref-67] Swofford DL (2002). PAUP* v40b10.

[ref-79] True FW (1905). Diagnosis of a new genus and species of fossil sea-lion from the Miocene of Oregon. Smithsonian Miscellaneous Collections.

[ref-68] Valenzuela-Toro AM, Pyenson ND, Gutstein CS, Suarez ME (2016). A new dwarf seal from the late Neogene of South America and the evolution of pinnipeds in the southern hemisphere. Papers in Palaeontology.

[ref-69] Valenzuela-Toro AM, Gutstein CS, Varas-Malca RM, Suarez ME, Pyenson ND (2013). Pinniped turnover in the South Pacific Ocean: new evidence from the Plio-Pleistocene of the Atacama Desert, Chile. Journal of Vertebrate Paleontology.

[ref-70] Vendrasco MJ, Eernisse DJ, Powell CLII, Fernandez CZ (2012). Polyplacophora (Mollusca) from the San Diego Formation: a remarkable assemblage of fossil chitins from the Pliocene of Southern California. Contributions in Science.

[ref-71] Vermeij GJ (2011). Shifting sources of productivity in the coastal marine tropics during the Cenozoic era. Proceedings of the Royal Society B: Biological Sciences.

[ref-72] Vermeij GJ (2012). The evolution of gigantism on temperate seashores. Biological Journal of the Linnean Society.

[ref-73] von Schreber JDC (1776). Die Säugethiere in Abbildungen nach der Natur, mit Beschreibungen. Part III.

[ref-74] Wagner HM, Riney BO, Deméré TA, Prothero DR (2001). Magnetic stratigraphy and land mammal biochronology of a nonmarine facies of the Pliocene San Diego Formation, San Diego County, California. SEPM, Special Publication.

[ref-75] Whistler DP, Lander EB (2003). New late Uintan to early Hemmingfordian land mammal assemblages from the undifferentiated Sespe and Vaqueros formations, Orange County, and from the Sespe and equivalent marine formations in Los Angeles, Santa Barbara, and Ventura counties, southern California. Bulletin of the American Museum of Natural History.

[ref-76] Yonezawa T, Kohno N, Hasegawa M (2009). The monophyletic origin of sea lions and fur seals (Carnivora; Otariidae) in the Southern Hemisphere. Gene.

