# Supplementary material for: Eotaria citrica, sp. nov., a new stem otariid from the “Topanga” formation of Southern California
Source: PeerJ. 2017 Feb 23;5:e3022. doi: 10.7717/peerj.3022 (PMC5326546; doi:10.7717/peerj.3022)
Supplement: Supplemental Information 2 [file peerj-05-3022-s002.docx]

| **Table S1.** Measurements (in mm) and morphological features observed for sexual dimorphism and intraspecific variation. | | | | | | | | |
| --- | --- | --- | --- | --- | --- | --- | --- | --- |
| Taxon | Ramus width @p4 | Ramus height @p4 | | Total length | | Diameter of canine | Sex | Position of genial tuberosity |
| *Eotaria crypta* | | | | | | | | |
| LACM 159981 | 4.92 | 14.10 | | 95.76 | | 4.96 | Unknown | p2 |
| *Eotaria citrica* n. sp. | | | | | | | | |
| LACM 122666 | 9.14 | 20.26 | | 115.10 | | 6.82 | Unknown | p2-3 |
| *Pithanotaria starri* | | | | | | | | |
| LACM uncat. | 7.64 | 19.32 | | 119.9 | | 5.92 | Unknown | p3 |
| LACM 22445 | 7.12 | 20.12 | | - | | - | Unknown | p3 |
| LACM 31202 | 8.16 | 21.88 | | - | | - | Unknown | NP |
| LACM 37582 | 8.36 | 21.02 | | - | | 7.84 | Unknown | p3 |
| LACM 115153 | 8.22 | 21.08 | | 127.76 | | 7.30 | Unknown | p3 |
| LACM 115677 | 6.20 | 20.06 | | 128.72 | | 5.94 | Unknown | p3 |
| LACM 117687 | 7.70 | 19.76 | | - | | - | Unknown | p3 |
| LACM 122620 | 9.84 | 22.5 | | - | | 5.42 | Unknown | p3 |
| LACM 122621 | 6.16 | 17.76 | | - | | - | Unknown | NP |
| Average | 7.71 | 20.39 | | 125.46 | | 6.48 |  |  |
| Standard deviation | 1.14 | 1.42 | | 4.84 | | 1.03 |  |  |
| *Zalophus californianus* | | | | | | | | |
| LACM 343 | 17.44 | 41.26 | 211.54 | | | 11.92 | Male | p2-3 |
| LACM 8584 | 8.72 | 23.84 | 158.24 | | | 6.26 | Female | p2-3 |
| LACM 8585 | 16.10 | 35.42 | 197.32 | | | 12.28 | Male | p2-3 |
| LACM 9337 | 16.12 | 39.72 | 216.66 | | | 11.20 | Male | p2-3 |
| LACM 22557 | 8.58 | 23.10 | 157.74 | | | 6.26 | Female | p2-3 |
| LACM 22999 | 8.66 | 22.18 | 152.9 | | | 6.16 | Unknown | p2-3 |
| LACM 23000 | 7.88 | 23.46 | 156.06 | | | 5.70 | Female | p2-3 |
| LACM 31275 | 14.88 | 37.52 | 204.48 | | | 12.30 | Male | p3 |
| LACM 31360 | 14.48 | 31.76 | 194.02 | | | 11.90 | Male | p1-2 |
| LACM 39652 | 11.98 | 31.10 | 193.62 | | | 10.56 | Male | p2-3 |
| LACM 39653 | 13.30 | 36.42 | 213.82 | | | 11.54 | Unknown | p2-3 |
| LACM 39654 | 9.72 | 26.6 | 158.64 | | | 9.18 | Male* | p2-3 |
| LACM 39655 | 13.76 | 38.52 | 203.92 | | | 12.62 | Male | p2-3 |
| LACM 39661 | 7.14 | 15.70 | 103.90 | | | 5.88 | Female* | p2-3 |
| LACM 39662 | 15.42 | 35.08 | 194.32 | | | 10.66 | Male | p2-3 |
| LACM 39663 | 14.02 | 37.58 | 189.16 | | | 10.58 | Male | p2-3 |
| LACM 39664 | 10.10 | 29.40 | 173.60 | | | 10.34 | Male | p2-3 |
| LACM 39665 | 17.08 | 37.68 | 205.06 | | | 12.04 | Male | p2-3 |
| LACM 39666 | 15.36 | 36.58 | 206.22 | | | 11.82 | Male | p2-3 |
| LACM 43482 | 15.72 | 39.56 | 195.74 | | | 11.94 | Male | p2-3 |
| LACM 51164 | 15.12 | 37.48 | 204.46 | | | 11.46 | Male | p2-3 |
| LACM 51170 | 21.48 | 41.6 | 216.36 | | | 12.54 | Male | p2-3 |
| LACM 51171 | 11.14 | 33.32 | 172.02 | | | 9.34 | Female | p2-3 |
| LACM 51175 | 11.26 | 32.18 | 189.48 | | | 10.42 | Male | p2-3 |
| LACM 51182 | 12.84 | 34.44 | 194.40 | | | 10.74 | Male | p2-3 |
| LACM 51191 | 14.82 | 37.20 | 198.56 | | | 11.16 | Male | p2-3 |
| LACM 51192 | 14.38 | 37.54 | 196.18 | | | 10.58 | Male | p2-3 |
| LACM 51197 | 7.52 | 24.9 | 147.04 | | | 6.38 | Unknown | p2-3 |
| LACM 51199 | 12.1 | 33.3 | 184.02 | | | 12.00 | Unknown | p2-3 |
| LACM 51204 | 10.74 | 30.82 | 180.58 | | | 10.50 | Male | p2-3 |
| LACM 51218 | 6.76 | 16.4 | 102.52 | | | 4.94 | Female* | p2-3 |
| LACM 51220 | 5.98 | 16.60 | 107.32 | | | 6.14 | Female* | p2-3 |
| LACM 51221 | 7.12 | 16.08 | 113.22 | | | 5.56 | Female* | p2-3 |
| LACM 51223 | 7.50 | 22.40 | 131.74 | | | 5.42 | Female | p2-3 |
| LACM 51228 | 8.82 | 22.54 | 147.18 | | | 5.76 | Female | p2-3 |
| LACM 51229 | 8.52 | 24.24 | 151.64 | | | 6.2 | Female | p2-3 |
| LACM 51237 | 15.74 | 40.00 | 188.30 | | | 13.04 | Male | p2-3 |
| LACM 52321 | 8.94 | 24.44 | 148.86 | | | 6.54 | Female | p2-3 |
| LACM 52406 | 8.56 | 25.86 | 159.72 | | | 6.76 | Female | p2-3 |
| LACM 52411 | 10.50 | 28.54 | 180.44 | | | 10.52 | Unknown | p2-3 |
| LACM 52412 | 11.7 | 29.84 | 176.70 | | | 10.80 | Male | p2-3 |
| LACM 52418 | 14.06 | 37.70 | 196.58 | | | 11.62 | Male | p2-3 |
| LACM 54104 | 11.72 | 35.94 | 182.78 | | | 11.52 | Unknown | p2-3 |
| LACM 54421 | 14.60 | 37.62 | 192.68 | | | 12.10 | Male | p2-3 |
| LACM 54462 | 9.14 | 26.58 | 157.46 | | | 9.74 | Male* | p2-3 |
| LACM 54578 | 6.18 | 23.40 | 148.20 | | | 6.80 | Unknown | p2-3 |
| LACM 54590 | 13.12 | 32.96 | 177.98 | | | 10.96 | Male | p2-3 |
| LACM 54624 | 12.38 | 32.60 | 192.32 | | | 12.40 | Male | p2-3 |
| LACM 84098 | 7.64 | 20.70 | 135.92 | | | 8.58 | Male* | p2-3 |
| LACM 86060 | 12.60 | 33.96 | 183.72 | | | 10.70 | Male | p2-3 |
| LACM 91326 | 8.26 | 23.18 | 145.9 | | | 6.42 | Female | p2-3 |
| LACM 91237 | 6.46 | 19.10 | 127.54 | | | 5.34 | Female | p2-3 |
| LACM 91328 | 10.14 | 25.80 | 162.36 | | | 8.98 | Male | p2-3 |
| LACM 91329 | 11.52 | 26.90 | 172.62 | | | 10.16 | Male | p2-3 |
| LACM 91332 | 7.96 | 26.06 | 143.00 | | | 5.70 | Female | p2-3 |
| LACM 91334 | 7.28 | 19.10 | 126.40 | | | 6.32 | Female | p2-3 |
| LACM 91761 | 8.46 | 25.34 | 146.16 | | | 6.68 | Female | p2-3 |
| LACM 91857 | 7.10 | 17.00 | 112.52 | | | 5.58 | Female* | p2-3 |
| LACM 91889 | 7.20 | 25.12 | 153.10 | | | 5.68 | Female | p2-3 |
| LACM 97236 | 8.36 | 23.82 | 153.98 | | | 6.24 | Female | p2-3 |
| LACM 97240 | 7.94 | 24.50 | 128.72 | | | 6.32 | Female | p2-3 |
| LACM 97517 | 7.32 | 24.06 | 155.80 | | | 6.44 | Female | p2-3 |
| LACM 97520 | 8.12 | 22.68 | 144.62 | | | 5.84 | Female | p2-3 |
| LACM 97569 | 8.58 | 23.44 | 144.18 | | | 5.62 | Female | p2-3 |
| LACM 97576 | 7.84 | 26.74 | 153.40 | | | 6.36 | Female | p2-3 |
| LACM 97578 | 8.02 | 22.98 | 151.94 | | | 5.98 | Female | p2-3 |
| LACM 97581 | 7.22 | 23.02 | 146.98 | | | 5.78 | Female | p2-3 |
| LACM 97586 | 12.08 | 32.06 | 186.72 | | | 9.92 | Male | p2-3 |
| LACM 97588  LACM 97597  Average | 11.94 | 30.16 | 173.40 | | | 9.88 | Male | p2-3 |
|  | 11.70 | 28.18 | 174.34 | | | 9.54 | Male | p2-3 |
|  | 10.90 | 28.90 | 167.86 | | | 8.87 |  |  |
| Standard deviation | 3.39 | 6.98 | 28.86 | | | 2.59 |  |  |
| *Callorhinus ursinus* | | | | | | | | |
| LACM 51351 | 7.58 | 16.96 | 114.00 | | | 5.78 | Female* | p2 |
| LACM 51353 | 7.62 | 16.72 | 113.28 | | | 5.52 | Female | p2 |
| LACM 51354 | 7.98 | 20.30 | 126.00 | | | 5.08 | Female | p2 |
| LACM 51356 | 7.94 | 18.44 | 121.96 | | | 6.10 | Female | p2 |
| LACM 51357 | 16.46 | 29.22 | 156.08 | | | 10.04 | Male | p2 |
| LACM 51545 | 5.68 | 14.22 | 110.28 | | | 5.56 | Unknown | p2 |
| LACM 52331 | 9.28 | 19.56 | 122.28 | | | 7.98 | Male* | p2 |
| LACM 52341 | 7.30 | 16.70 | 115.14 | | | 5.16 | Female | p2 |
| LACM 52342 | 10.86 | 27.48 | 145.62 | | | 8.10 | Unknown | p2 |
| LACM 86090  Average  Standard deviation | 6.00 | 13.44 | 90.1 | | | 5.08 | Female* | p2 |
|  | 8.67 | 19.30 | 121.47 | | | 6.44 |  |  |
|  | 3.11 | 5.23 | 18.48 | | | 1.69 |  |  |
| *Eumetopias jubatus* | | | | | | | | |
| LACM 616 | 25.32 | 62.76 | 297.18 | | | 20.10 | Male | p1-2 |
| LACM 620 | 11.78 | 36.12 | 219.22 | | | 9.58 | Male | p2 |
| LACM 21443 | 11.04 | 31.82 | 185.62 | | | 8.38 | Unknown | p2 |
| LACM 39651 | 11.78 | 37.72 | 204.50 | | | 10.04 | Female | p1-2 |
| LACM 51173 | 12.74 | 37.62 | 219.66 | | | 9.22 | Unknown | p2-3 |
| LACM 51232 | 14.98 | 40.08 | 216.08 | | | 10.52 | Female | p1-2 |
| LACM 52311 | 12.52 | 40.42 | 225.50 | | | 9.94 | Female | 1p2 |
| LACM 52312 | 17.80 | 46.96 | 255.64 | | | 16.87 | Male | p1-2 |
| LACM 52313 | 11.62 | 34.46 | 214.92 | | | 9.60 | Female | p1-2 |
| LACM 52315 | 13.04 | 36.44 | 217.08 | | | 8.08 | Unknown | p2 |
| LACM 97334  Average  Standard deviation | 10.20 | 37.24 | 204.18 | | | 10.26 | Female | p2 |
|  | 13.89 | 40.15 | 223.60 | | | 11.14 |  |  |
|  | 4.32 | 8.43 | 29.73 | | | 3.76 |  |  |
| Ratios between ramus width/height @p4 | | | | | Average | | Standard deviation | |
| *Eotaria crypta* | | | | | 0.35^1^ | | ^-^ | |
| *Eotaria citrica* n. sp. | | | | | 0.45^1^ | | ^-^ | |
| *Pithanotaria starri* | | | | | 0.38 | | 0.04 | |
| *Zalophus californianus* female | | | | | 0.34 | | 0.03 | |
| *Zalophus californianus* male | | | | | 0.39 | | 0.04 | |
| *Callorhinus ursinus* female | | | | | 0.43 | | 0.02 | |
| *Callorhinus ursinus* male | | | | | 0.51 | | 0.06 | |
| *Eumetopias jubatus* female | | | | | 0.32 | | 0.04 | |
| *Eumetopias jubatus* male | | | | | 0.37 | | 0.04 | |
| *juvenile specimens; ^1^actual ratio on single specimen; NP = not preserved. | | | | | | | | |
