# Supplementary material for: Eotaria citrica, sp. nov., a new stem otariid from the “Topanga” formation of Southern California
Source: PeerJ. 2017 Feb 23;5:e3022. doi: 10.7717/peerj.3022 (PMC5326546; doi:10.7717/peerj.3022)
Supplement: Supplemental Information 3 [file peerj-05-3022-s003.docx]

| **Table S2.** Loadings of PC1 and PC2. | | |  |  |  |  |  |  |
| --- | --- | --- | --- | --- | --- | --- | --- | --- |
|  | PC1 | PC2 |  |  |  |  |  |  |
| Diameter | 0.501 | 0.263 |  |  |  |  |  |  |
| Length | 0.499 | -0.620 |  |  |  |  |  |  |
| Height | 0.507 | -0.305 |  |  |  |  |  |  |
| Width | 0.493 | 0.673 |  |  |  |  |  |  |
|  | | |  |  |  |  |  |  |
