# Supplementary material for: Eotaria citrica, sp. nov., a new stem otariid from the “Topanga” formation of Southern California
Source: PeerJ. 2017 Feb 23;5:e3022. doi: 10.7717/peerj.3022 (PMC5326546; doi:10.7717/peerj.3022)
Supplement: Supplemental Information 4 [file peerj-05-3022-s004.docx]

| **Table S3.** Distance between medoids of each cluster of species included in the principal component analysis. | | | | | | | | |
| --- | --- | --- | --- | --- | --- | --- | --- | --- |
|  | *E. cr.* | *E. cit.* | *Z. cal.* (f) | *Z. cal.* (m) | *C. urs.* (f) | *C. urs.* (m) | *E. jub.* (f) | *E. jub.* (m) |
| *E. cr.* | 0 | 1.5143 | 1.9807 | 4.7801 | 0.9031 | 4.1261 | 4.7377 | 10.7576 |
| *E. cit.* | 1.5143 | 0 | 1.0654 | 3.3678 | 0.6217 | 2.6120 | 3.4455 | 9.3024 |
| *Z. cal.* (f) | 1.9807 | 1.0654 | 0 | 2.9055 | 1.2403 | 2.6395 | 2.7746 | 8.8847 |
| *Z. cal.* (m) | 4.7801 | 3.3678 | 2.9055 | 0 | 3.9014 | 1.4447 | 0.7217 | 5.9868 |
| *C. urs.* (f) | 0.9031 | 0.6217 | 1.2403 | 3.9014 | 0 | 3.2268 | 3.9060 | 9.8676 |
| *C. urs.* (m) | 4.1261 | 2.6120 | 2.6395 | 1.4447 | 3.2268 | 0 | 2.0351 | 6.8277 |
| *E. jub.* (f) | 4.7377 | 3.4455 | 2.7746 | 0.7217 | 3.9060 | 2.0351 | 0 | 6.1833 |
| *E. jub.* (m) | 10.7576 | 9.3024 | 8.8847 | 5.9868 | 9.8676 | 6.8277 | 6.1833 | 0 |
| Abbreviations: *C. urs.* = *Callorhinus ursinus*; *E. cit.* = *Eotaria citrica*; *E. cr.* = *Eotaria crypta*; *E. jub.* = *Eumetopias jubatus*; f = female; m = male; *Z. cal.* = *Zalophus californianus*. | | | | | | | | |
