# Supplementary material for: Eotaria citrica, sp. nov., a new stem otariid from the “Topanga” formation of Southern California
Source: PeerJ. 2017 Feb 23;5:e3022. doi: 10.7717/peerj.3022 (PMC5326546; doi:10.7717/peerj.3022)
Supplement: Supplemental Information 5 [file peerj-05-3022-s005.docx]

| Table S4. List of specimens used for body length estimates and their corresponding lengths based on equations from Churchill et a. (2015). | | |
| --- | --- | --- |
| Species | Specimens | Estimated body length (cm) |
| 1) *Pinnarctidion bishopi* | UCMP 86334 | 132^1^ |
| 2) *Enaliarctos mitchelli* | UCMP 80943 | 169^1^ |
| 3) *Enaliarctos mealsi* | USNM 374272, 4321 | 183^1^ |
| 4) *Eotaria crypta* | LACM 159981 | 125 |
| 5) *Eotaria citrica* | LACM 122666 | 140 |
| 6) *Neotherium* sp. | LACM uncat. | 218 |
| 7) *Pelagiarctos* sp. | SDNHM 131041 | 234^1^ |
| 8) *Allodesmus* sp. | LACM 126199; OCPC 5670 | 255 |
| 9) Pinnipedia indet. | LACM 127710 | 155 |
| 10) *Neotherium mirum* | LACM 81665, 123002, 131950, 134393 | 224 |
| 11) *Pelagiarctos thomasi* | LACM 121501 | 234^2^ |
| 12) *Allodesmus kelloggi* | LACM 4320 | 282^1^ |
| 13) *Allodesmus courseni* | LACM 1376 | 221 |
| 14) *Allodesmus* sp. | LACM 160016 | 308 |
| 15) *Pithanotaria starri* | LACM 22449, 115153, 115677 | 155 |
| 16) Odobenidae sp. 1 | LACM 123282 | 229 |
| 17) Odobenidae sp. 2 | LACM 122444 | 339 |
| 18) Odobenidae sp. 3 | LACM 4324, 17588 | 349 |
| 19) Pan-Otariidae sp. 1 | OCPC 1893, 1894 | 159 |
| 20) *Thalassoleon* sp. | LACM 128005, 150914 | 196 |
| 21) Odobenidae sp. 4 | LACM 118967 | 293 |
| 22) Odobenidae sp. 5 | LACM 150922; RMW SW90-40B | 307 |
| 23) *Gomphotaria pugnax* | LACM 121508; LC 7750 | 337 |
| 24) *Callorhinus gilmorei* | SDSNH 25716 | 157^1^ |
| 25) *Valenictus chulavistensis* | SDSNH 90497, 83719 | 218^1^ |
| 26) *Dusignathus seftoni* | SDSNH 83686, 83722 | 282^1^ |
| ^1^Data from Churchill et al., 2015; ^2^estimate based on *Pelagiarctos* sp. (SDNHM 131041). | | |
